# Supplementary material for: Phenotypic and Genotypic Identification of Dermatophytes from Mexico and Central American Countries
Source: J Fungi (Basel). 2023 Apr 11;9(4):462. doi: 10.3390/jof9040462 (PMC10143779; doi:10.3390/jof9040462)
Supplement: Supplementary file 1 [file jof-09-00462-s001.zip › Supplementary material-Figure S1.pdf]

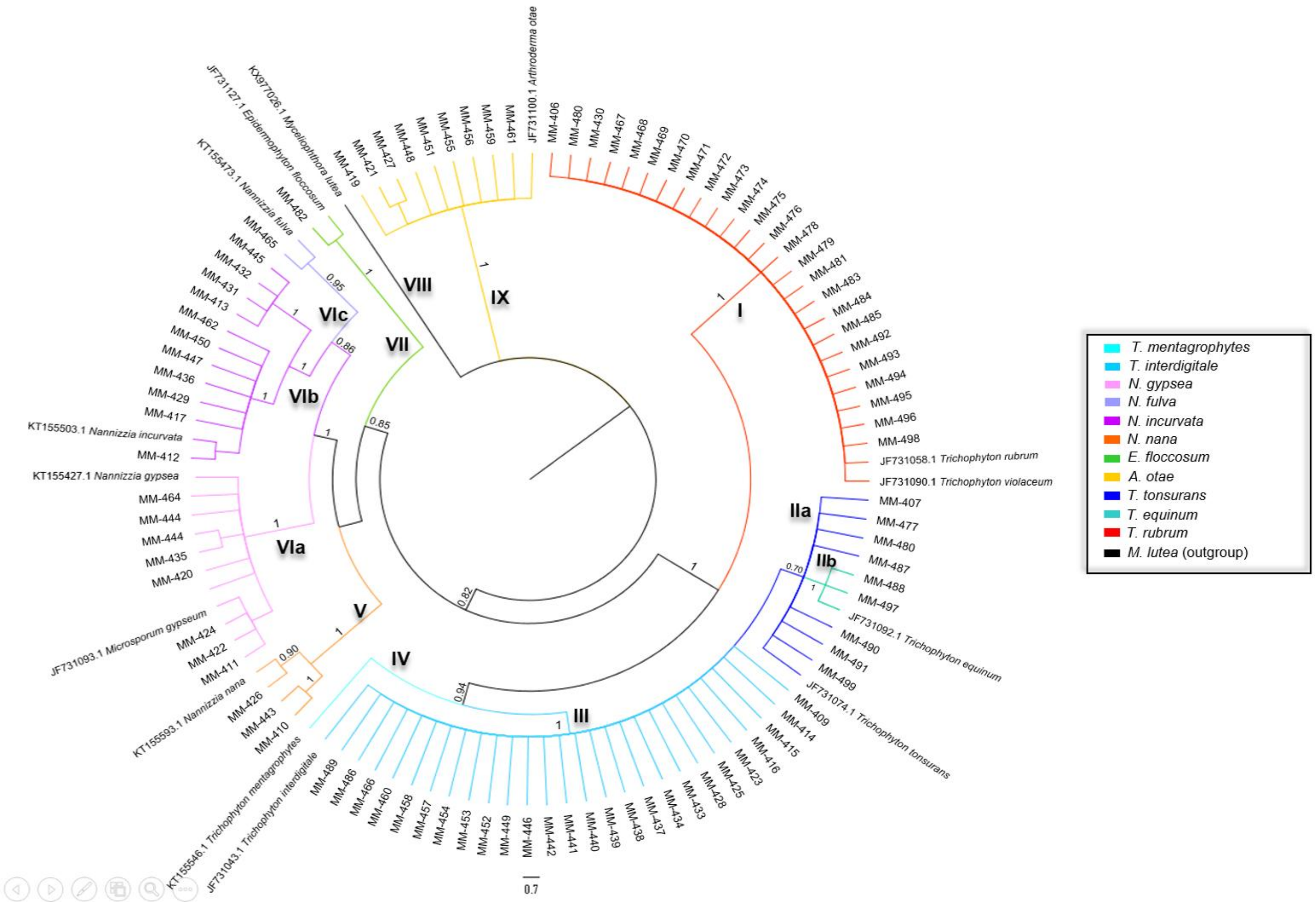

Supplementary Figure S1. Phylogenetic tree built with BT2 gene sequences using Bayesian inference, through the Mr. Bayes program. The supporting values of the posterior probability are shown in the nodes.
