# Supplementary material for: Phenotypic and Genotypic Identification of Dermatophytes from Mexico and Central American Countries
Source: J Fungi (Basel). 2023 Apr 11;9(4):462. doi: 10.3390/jof9040462 (PMC10143779; doi:10.3390/jof9040462)
Supplement: Supplementary file 1 [file jof-09-00462-s001.zip › Supplementary material-Figure S2.pdf]

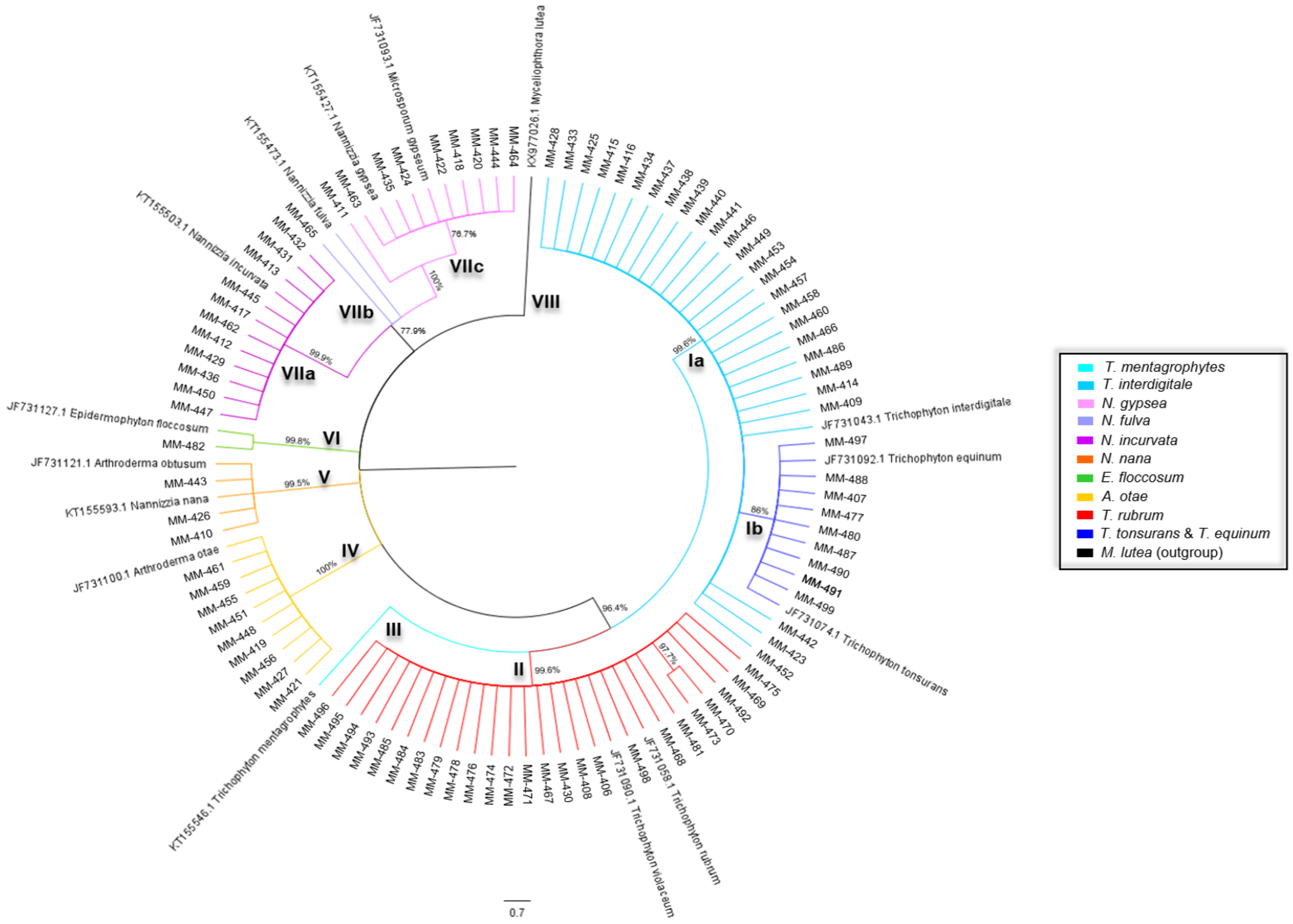

Supplementary Figure S2. Phylogenetic tree of dermatophytes built from the BT2 gene sequences, using the maximum likelihood method through the MEGAX program. Bootstrap support values are displayed in nodes.
