# Supplementary material for: Phenotypic and Genotypic Identification of Dermatophytes from Mexico and Central American Countries
Source: J Fungi (Basel). 2023 Apr 11;9(4):462. doi: 10.3390/jof9040462 (PMC10143779; doi:10.3390/jof9040462)
Supplement: Supplementary file 1 [file jof-09-00462-s001.zip › Supplementary material-Figure S3.pdf]

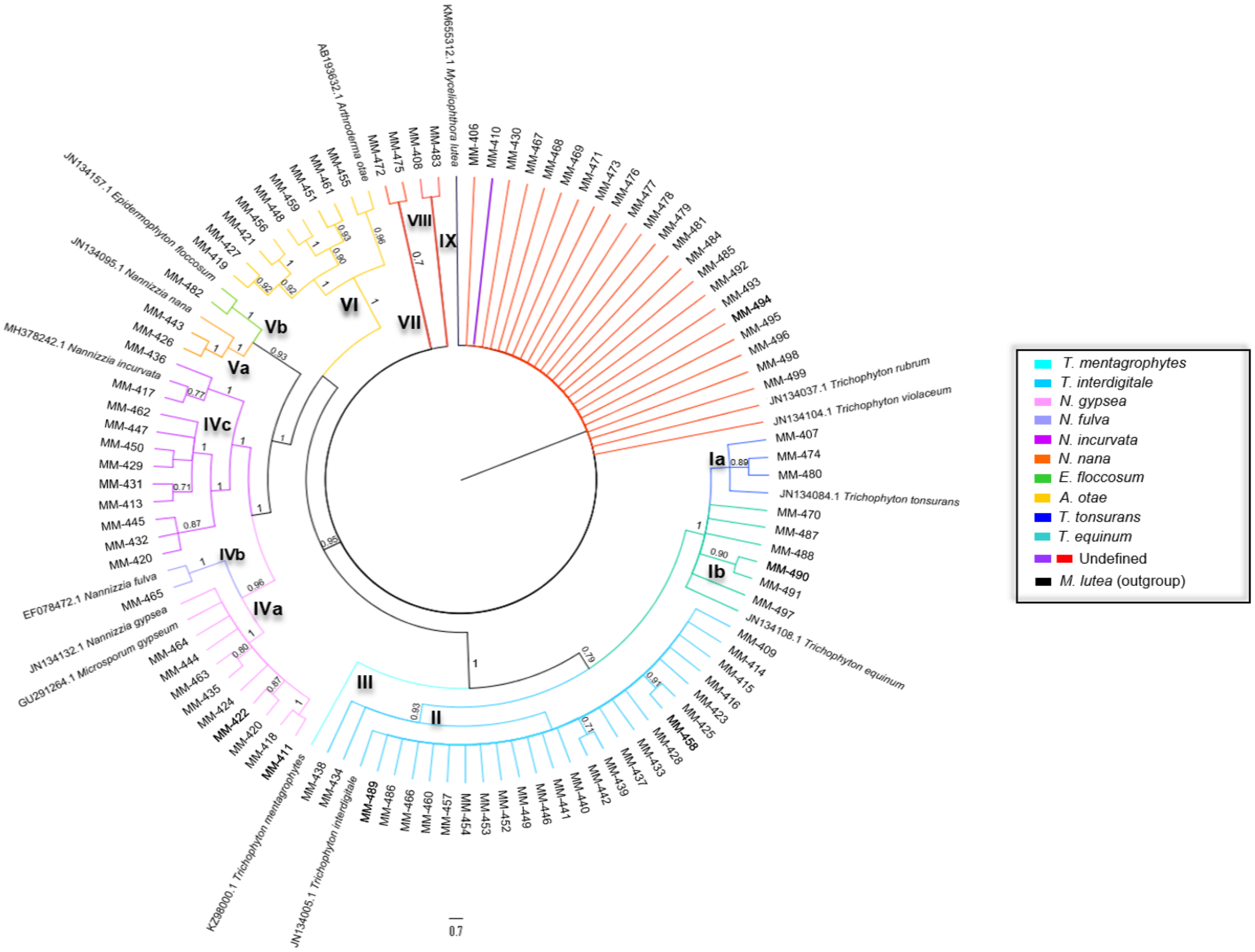

Supplementary Figure S3. Phylogenetic tree built with sequences of ITS region using Bayesian inference, through the Mr. Bayes program, from one million repetitions. The supporting values of the posterior probability are shown in the nodes.
