# Supplementary material for: Phenotypic and Genotypic Identification of Dermatophytes from Mexico and Central American Countries
Source: J Fungi (Basel). 2023 Apr 11;9(4):462. doi: 10.3390/jof9040462 (PMC10143779; doi:10.3390/jof9040462)
Supplement: Supplementary file 1 [file jof-09-00462-s001.zip › Supplementary material-Figure S5.pdf]

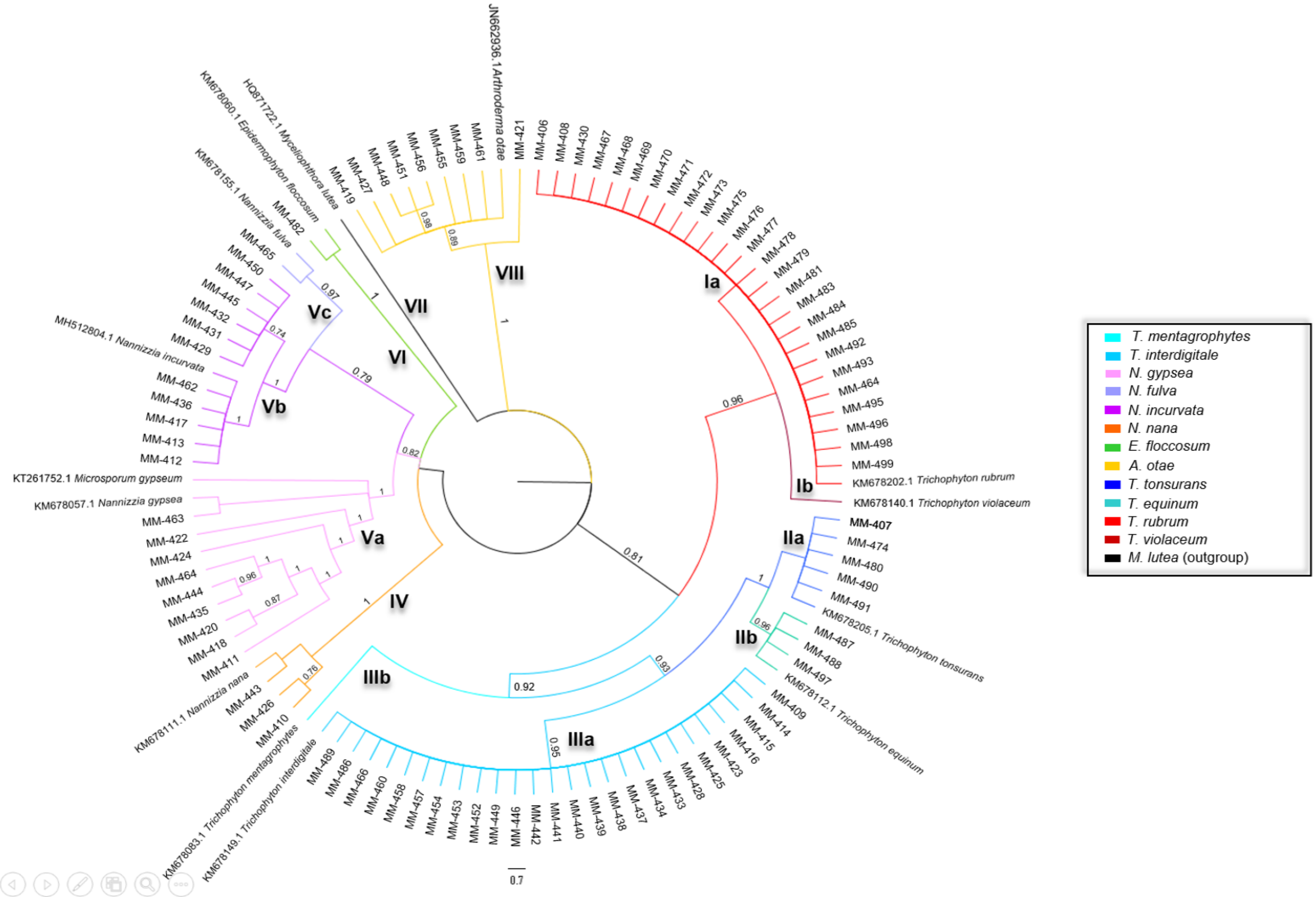

Supplementary Figure S5. Phylogenetic tree built with sequences of Tef1-α gene using the Bayesian inference, through the Mr. Bayes program, from one million repetitions. The support values of the posterior probability are shown in the nodes.
