# Supplementary material for: Phenotypic and Genotypic Identification of Dermatophytes from Mexico and Central American Countries
Source: J Fungi (Basel). 2023 Apr 11;9(4):462. doi: 10.3390/jof9040462 (PMC10143779; doi:10.3390/jof9040462)
Supplement: Supplementary file 1 [file jof-09-00462-s001.zip › Supplementary material-Figure S6.pdf]

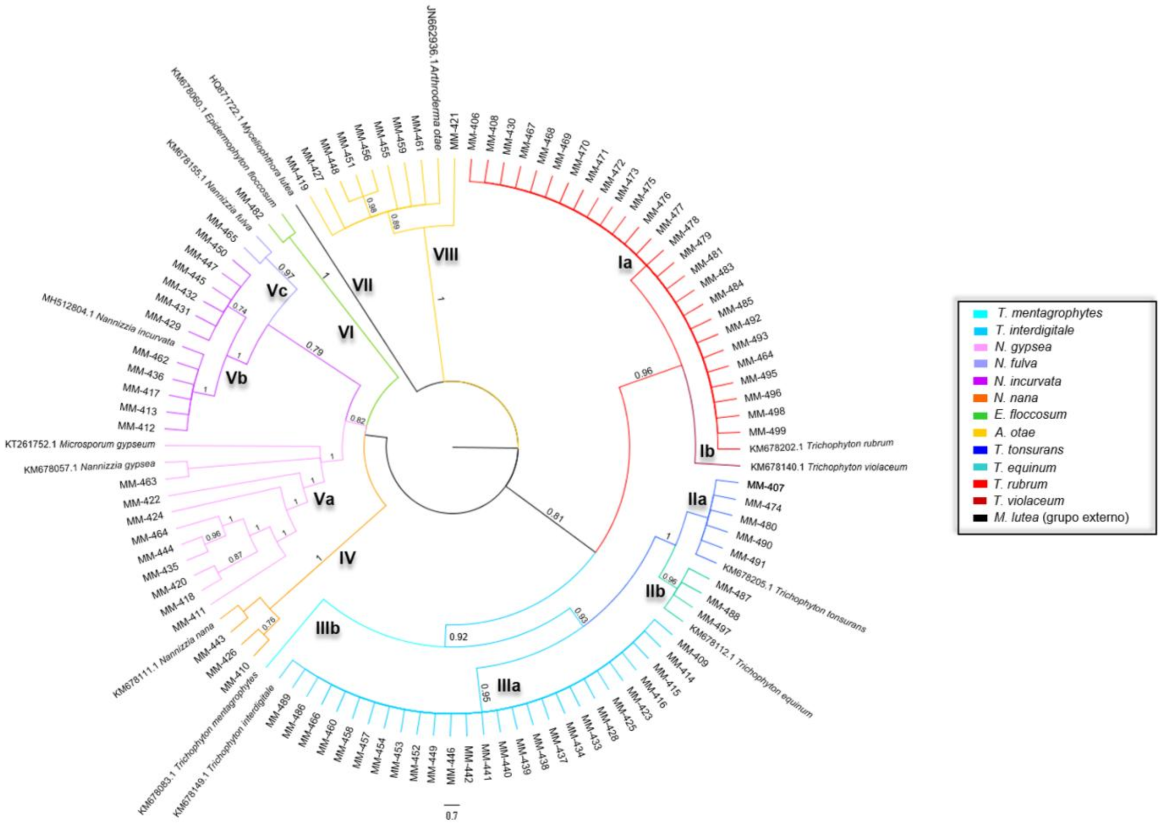

Supplementary Figure S6. Phylogenetic tree built with sequences of *Tef-1α* gene using maximum likelihood, trough the MEGAX program. Bootstrap support values are displayed in nodes.
