# Supplementary material for: Phenotypic and Genotypic Identification of Dermatophytes from Mexico and Central American Countries
Source: J Fungi (Basel). 2023 Apr 11;9(4):462. doi: 10.3390/jof9040462 (PMC10143779; doi:10.3390/jof9040462)
Supplement: Supplementary file 1 [file jof-09-00462-s001.zip › Supplementary material-Table S1.pdf]

Supplementary Table S1. Macro and micromorphological description of dermatophyte isolates included in the study.

	Macromorphology		Micromorphology
	Culture medium	Culture medium	
Isolate	Sabouraud agar with cycloheximide and chloramphenicol	Potato dextrose agar	
Trichophyton rubrum			
CR10	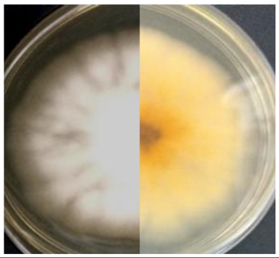	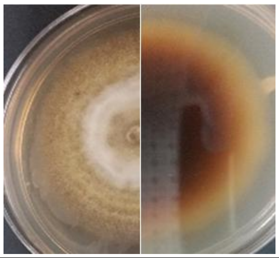	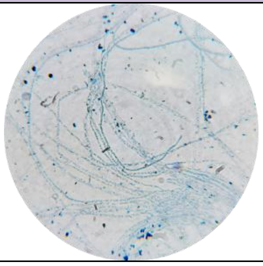
	**Obverse**: white colony, with a velvety surface, with radial growth, limited and regular border.<br><br>**Reverse**: production of yellow pigment, not diffusible in the medium.	**Obverse**: white-yellow colony, with a hairy surface, with radial growth, limited and regular border.<br><br>**Reverse**: production of red-brown pigment, not diffusible in the medium.	Coenocytic, sterile mycelium.
DO19	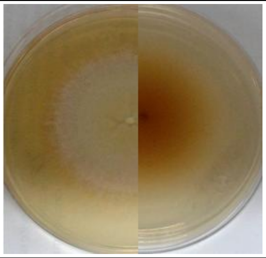	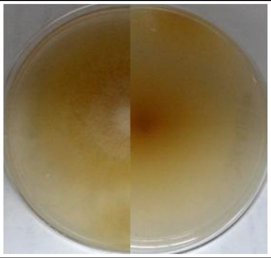	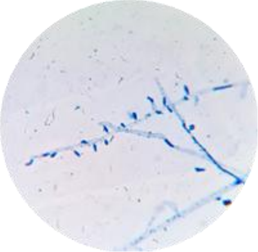
	**Obverse**: white-yellow colony, with a velvety surface, with radial growth, limited and regular border.<br><br>**Reverse**: production of yellow-orange pigment, not diffusible in the medium.	**Obverse**: white colony, with a velvety surface, with radial growth, limited and regular border.<br><br>**Reverse**: production of light brown pigment, not diffusible in the medium.	Septate mycelium, with abundant pyriform and round microconidia; free and arranged alternately throughout the mycelium.<br><br>**Microconidia size ($\bar{X}$):** 4.5 x 2.3µm
DO23	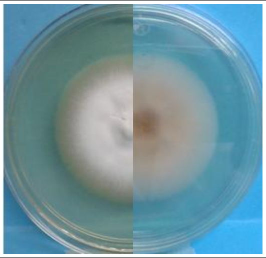	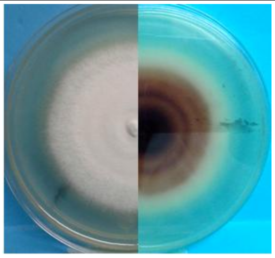	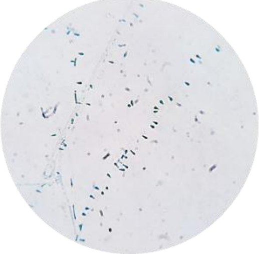
	**Obverse**: white colony, umbilicated, with a velvety	**Obverse**: white colony, with a hairy surface, with radial growth,	Septate mycelium, with abundant pyriform

	surface, with radial growth, limited and regular border.<br><br>**Reverse** pigment production absent.	limited and regular border.<br><br>**Reverse**: production of red-brown pigment, not diffusible in the medium.	microconidia; free and arranged alternately throughout the mycelium.<br><br>**Microconidia size** ($\bar{X}$): 3.6 x 1.9µm.
**GT25**	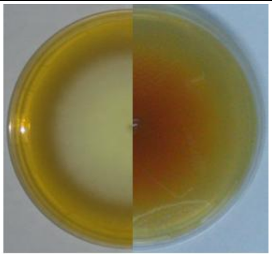	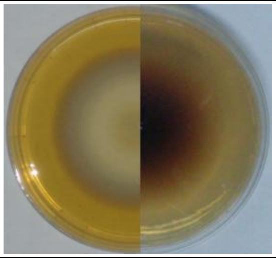	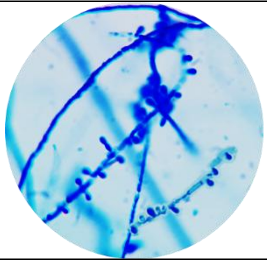
	**Obverse**: yellow colony, with velvety surface, with radial growth, limited and regular border.<br><br>**Reverse**: production of yellow-orange pigment, not diffusible in the medium.	**Obverse**: yellow-pink colony, with a hairy surface, with radial growth, limited and regular border.<br><br>**Reverse**: production of brown pigment, not diffusible in the medium.	Septate mycelium, with few pyriform microconidia arranged alternately along the mycelium.<br><br>**Microconidia size** ($\bar{X}$): 2.8 x 1.3µm.
**HN05**	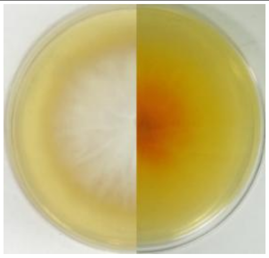	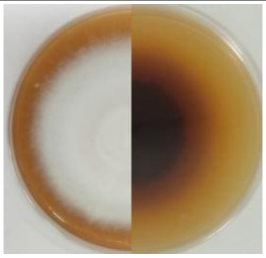	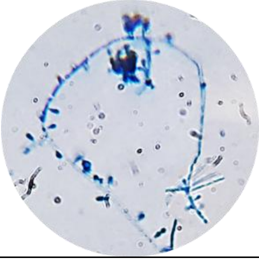
Descripción	**Obverse**: white colony, crateriform, with a velvety surface, with radial growth, limited and regular border.<br><br>**Reverse**: production of yellow-orange pigment, not diffusible in the medium.	**Obverse**: white-pink colony, cottony surface, with radial growth, limited and regular border.<br><br>**Reverse**: production of red-wine pigment, diffusible in the medium.	Septate mycelium, with few pyriform microconidia arranged along the mycelium; to a lesser extent located in the form of a "cross of Lorraine".<br><br>**Microconidia size** ($\bar{X}$): 2.5 x 1.2µm
**MX45**	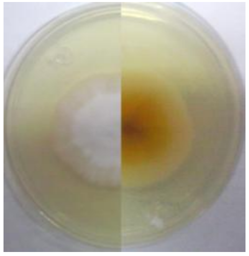	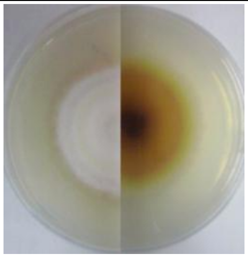	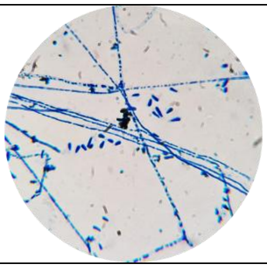

	**Obverse**: white colonies, with a velvety surface, with radial growth, limited and regular border.<br><br>**Reverse**: production of brown-yellow pigment, not diffusible in the medium.	**Obverse**: white colonies, with a hairy surface, with radial growth, limited and regular border.<br><br>**Reverse**: production of brown-yellow pigment, diffusible in the medium.	Septate mycelium, with few pyriform microconidia; free and arranged throughout the mycelium.<br><br>**Microconidia size** ($\bar{X}$): 3.8 x 1.7μm.
**MX47**	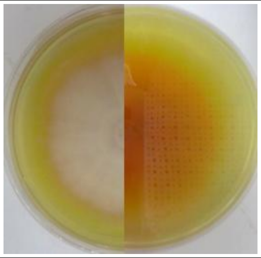	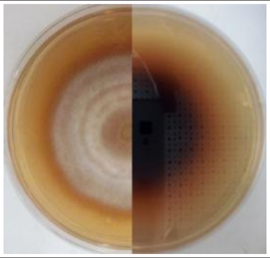	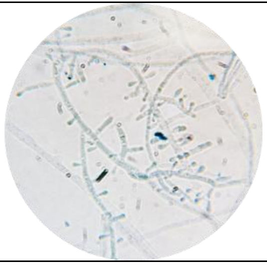
	**Obverse**: white colony, with a velvety surface, with radial growth, limited and regular border.<br><br>**Reverse**: production of yellow-orange pigment, diffusible in the medium.	**Obverse**: pale yellow colony, downy surface, radial growth, limited and regular border.<br><br>**Reverse**: production of red-wine pigment, diffusible in the medium.	Septate mycelium, with few pyriform microconidia; arranged throughout the mycelium.<br><br>**Microconidia size** ($\bar{X}$): 3.8 x 1.9μm.
**MX48**	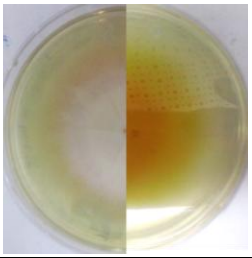	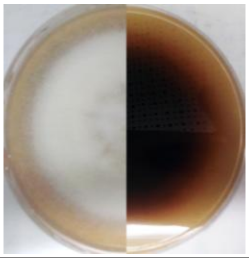	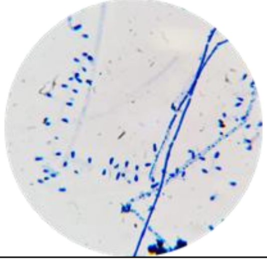
	**Obverse**: pale yellow colony, crater-shaped, with velvety surface, with radial growth, limited and regular border.<br><br>**Reverse**: production of yellow-orange pigment, not diffusible in the medium.	**Obverse**: white-pink colony, crater-shaped, with cottony surface, with radial growth and limited and regular border.<br><br>**Reverse**: production of red-wine pigment, diffusible in the medium.	Septate mycelium, with abundant pyriform microconidia; free and to a lesser extent arranged alternately throughout the mycelium.<br><br>**Microconidia size** ($\bar{X}$): 3.6 x 1.8μm.

**MX51**	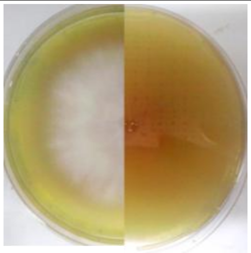	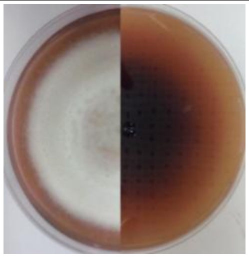	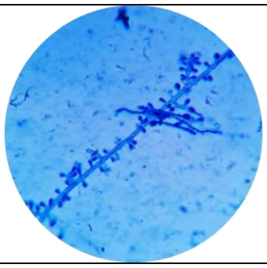
	**Obverse**: white colony, with a velvety surface, with radial growth, limited and regular border.<br><br>**Reverse**: production of yellow-orange pigment, diffusible in the medium.	**Obverse**: white-pink colony, with a hairy surface, radial growth, limited and regular border.<br><br>**Reverse**: production of red-wine pigment, diffusible in the medium.	Septate mycelium, with few pyriform microconidia; arranged parallel to the length of the mycelium.<br><br>**Microconidia size** ($\bar{X}$): 3.5 x 1.7μm.
**MX52**	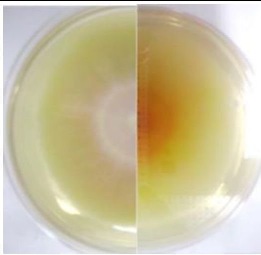	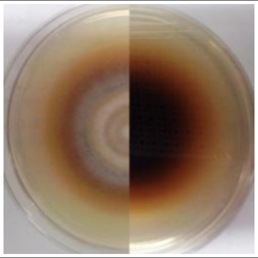	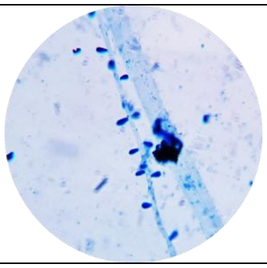
Descripción	**Obverse**: white colony, with a velvety surface, with radial growth, limited and regular border.<br><br>**Reverse**: production of yellow-orange pigment, not diffusible in the medium.	**Obverse**: yellow-pink colony, with a hairy surface, with radial growth, limited and regular border.<br><br>**Reverse**: production of red-wine pigment, diffusible in the medium.	Coenocytic mycelium, with few pyriform microconidia; free and arranged parallel to the length of the mycelium.<br><br>**Microconidia size** ($\bar{X}$): 3.5 x 1.7μm.
**MX53**	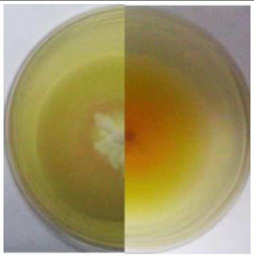	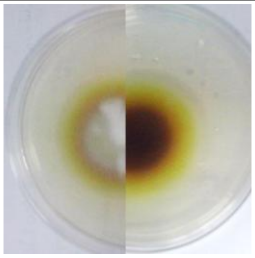	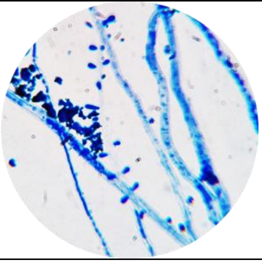
Descripción	**Obverse**: white colony, crateriform, with a velvety surface, with radial growth, limited and regular border.<br><br>**Reverse**: production of yellow-orange pigment, not diffusible in	**Obverse**: white-yellow colony, with a hairy surface, with radial growth, limited and regular border.<br><br>**Reverse**: production of brown-yellow pigment, not diffusible in	Septate mycelium, with few pyriform microconidia; arranged parallel to the length of the mycelium.<br><br>**Microconidia size** ($\bar{X}$): 3.9 x 2.0μm.

	the medium.	the medium.	
**MX56**	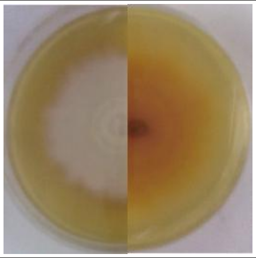	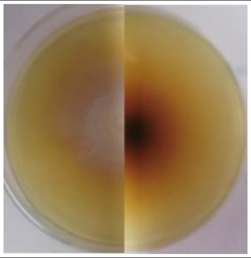	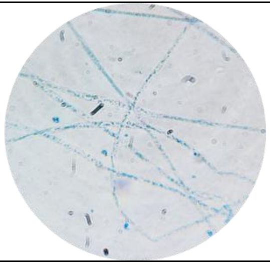
	**Obverse**: white colony, with a velvety surface, with radial growth, limited and regular border.<br><br>**Reverse**: production of yellow-orange pigment, not diffusible in the medium.	**Obverse**: white colony, with a velvety surface, with radial growth, limited and regular border.<br><br>**Reverse**: production of brown-yellow pigment, diffusible in the medium.	Coenocytic, sterile mycelium.
**MX58**	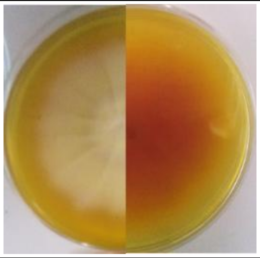	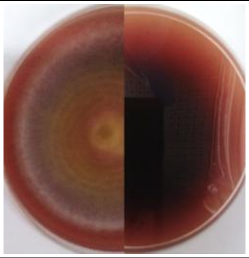	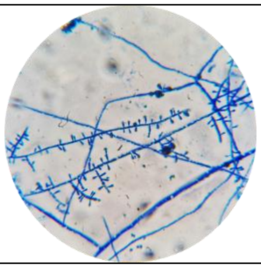
	**Obverse**: pale yellow colony, crater-shaped, with a velvety surface, with radial growth and a limited and regular border.<br><br>**Reverse**: production of yellow-orange pigment, diffusible in the medium.	**Obverse**: pale pink-green-yellow colony, with a hairy surface, with radial growth, limited and regular border.<br><br>**Reverse**: production of red-wine pigment, diffusible in the medium.	Septate mycelium with abundant pyriform microconidia; free and arranged parallel throughout the mycelium; to a lesser extent, located in the shape of a "Lorraine cross".<br><br>**Microconidia size** ($\bar{X}$): 3.7 x 1.4μm.
**MX59**	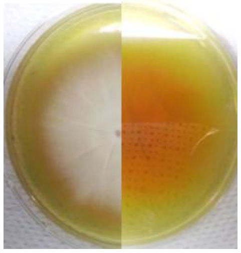	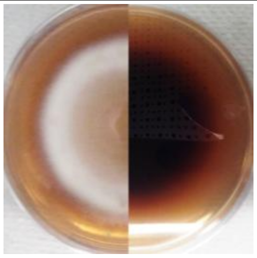	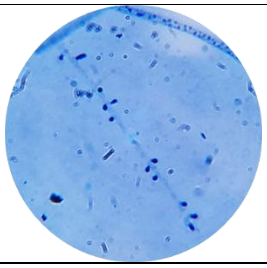
	**Obverse**: pale yellow colony, crater-shaped, with a velvety surface, with radial growth and a limited and regular border.<br><br>**Reverse**: production of yellow-	**Obverse**: pale yellow colony, cottony surface, with radial growth, limited and regular border.<br><br>**Reverse**: production of red-wine	Coenocytic mycelium with few pyriform microconidia; arranged throughout the mycelium.<br><br>**Microconidia size** ($\bar{X}$): 3.4 x

	orange pigment, not diffusible in the medium.	pigment, diffusible in the medium.	1.7μm.
**MX63**	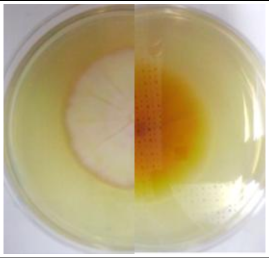	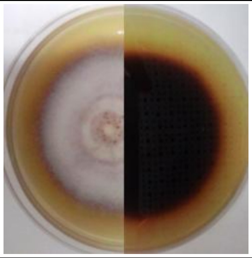	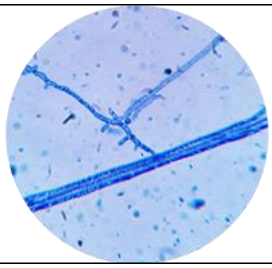
Descripción	**Obverse**: pale yellow colony, crater-shaped, with a velvety surface, with radial growth and a limited and regular border.<br><br>**Reverse**: production of yellow-orange pigment, diffusible in the medium.	**Obverse**: white-pink colony, crateriform, umbilicate, with a hairy surface, with radial growth and limited and regular border, production of metabolites.<br><br>**Reverse**: production of red-wine pigment, diffusible in the medium.	Coenocytic mycelium, with few pyriform microconidia; arranged throughout the mycelium.<br><br>**Microconidia size** ($\bar{X}$): 3.5 x 1.5μm.
**MX66**	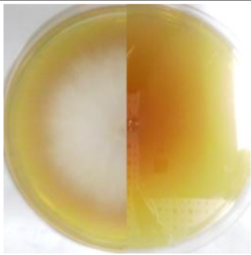	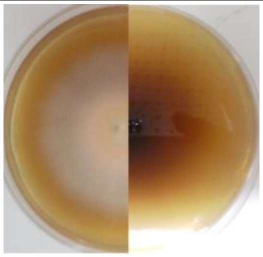	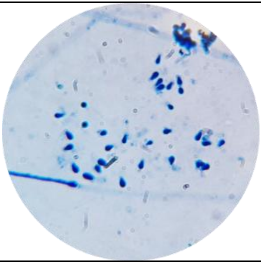
	**Obverse**: white colony, with a velvety surface, with radial growth, limited and regular border.<br><br>**Reverse**: production of yellow-orange pigment, diffusible in the medium.	**Obverse**: pale pink colony, velvety surface, radial growth, limited and regular border.<br><br>**Reverse**: production of brown-yellow pigment, diffusible in the medium.	Coenocytic mycelium with few pyriform microconidia; free and arranged throughout the mycelium.<br><br>**Microconidia size** ($\bar{X}$): 3.7 x 1.6μm.
**MX70**	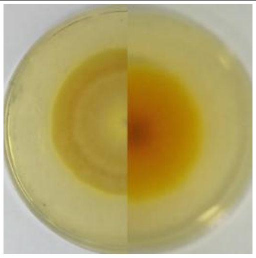	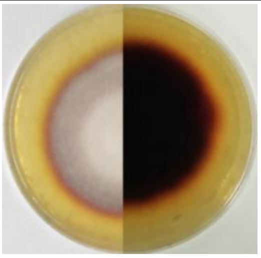	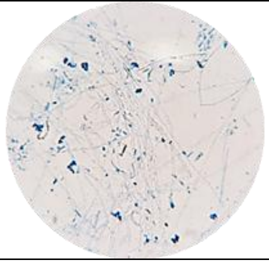
	**Obverse**: pale yellow colony, velvety surface, radial growth,	**Obverse**: white-pink colony, with a velvety surface, with radial	Coenocytic mycelium with abundant free pyriform

	limited and regular border.<br><br>**Reverse**: production of yellow-orange pigment, not diffusible in the medium.	growth, limited and regular border.<br><br>**Reverse**: production of red-wine pigment, diffusible in the medium.	microconidia; to a lesser extent arranged along the mycelium.<br><br>**Microconidia size** (X̅): 3.5 x 1.5µm.
**MX73**	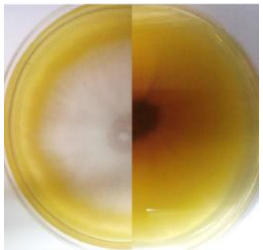	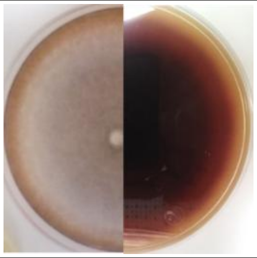	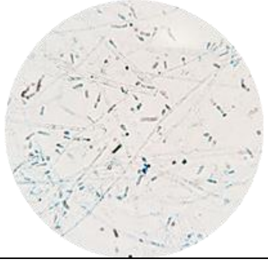
	**Obverse**: white colony, crateriform, with a velvety surface, with radial growth and a limited and regular border.<br><br>**Reverse**: production of brown-yellow pigment, not diffusible in the medium.	**Obverse**: pale pink colony, velvety surface, radial growth, limited and regular border.<br><br>**Reverse**: production of red-wine pigment, diffusible in the medium.	Coenocytic mycelium with abundant pyriform microconidia; free and arranged throughout the mycelium.<br><br>**Microconidia size** (X̅): 3.7 x 1.4µm.
**MX74**	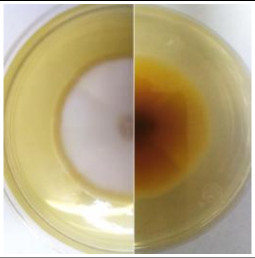	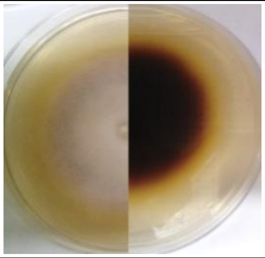	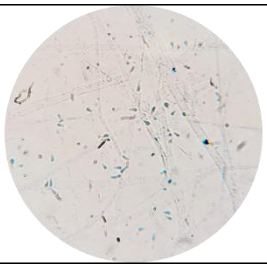
	**Obverse**: white colony, crateriform, with a velvety surface, with radial growth and a limited and regular border.<br><br>**Reverse**: production of brown-yellow pigment, not diffusible in the médium.	**Obverse**: white-pink colony, with velvety surface, with radial growth, limited and regular border.<br><br>**Reverse**: production of red-wine pigment, diffusible in the medium.	Coenocytic mycelium with abundant pyriform microconidia; arranged throughout the mycelium.<br><br>**Microconidia size** (X̅): 3.0 x 1.6µm.
**MX78**	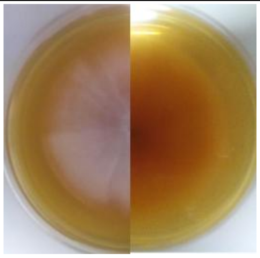	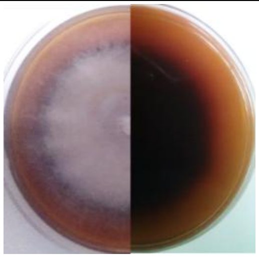	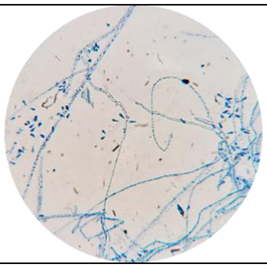

	**Obverse**: pale pink colony, crateriform, with velvety surface, with radial growth and limited and regular border.<br><br>**Reverse**: production of yellow-orange pigment, diffusible in the médium.	**Obverse**: pale pink colony, downy surface, radial growth, limited and regular border.<br><br>**Reverse**: production of red-wine pigment, diffusible in the medium.	Coenocytic mycelium with abundant pyriform microconidia; arranged throughout the mycelium.<br><br>**Microconidia size** ($\bar{X}$): 3.7 x 1.6µm.
**MX87**	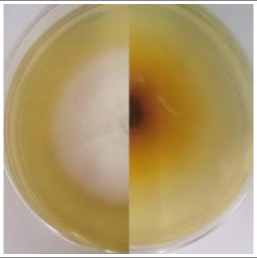	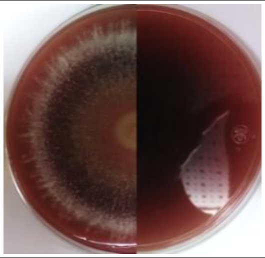	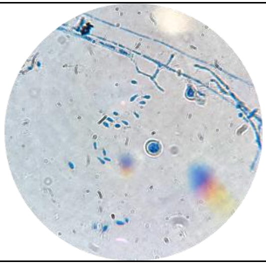
	**Obverse**: white colony, with a velvety surface, with radial growth, limited and regular border.<br><br>**Reverse**: production of brown-yellow pigment, not diffusible in the medium.	**Obverse**: pale pink-green-yellow colony, with a hairy surface, with radial growth, limited and regular border.<br><br>**Reverse**: production of red-wine pigment, diffusible in the medium.	Septate mycelium with abundant pyriform microconidia; free and arranged throughout the mycelium.<br><br>**Microconidia size** ($\bar{X}$): 4.0 x 2.0µm.
**MX89**	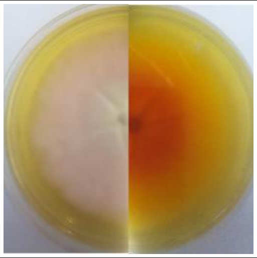	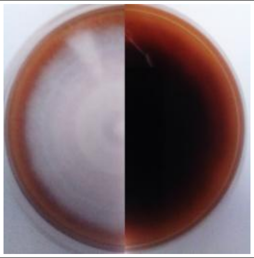	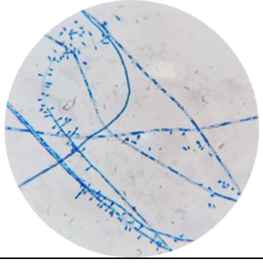
	**Obverse**: pale yellow-pink colony, crater-shaped, with a velvety surface, with radial growth and a limited and regular border.<br><br>**Reverse**: production of yellow-orange pigment, diffusible in the medium.	**Obverse**: white-pink colony, with velvety surface, with radial growth, limited and regular border.<br><br>**Reverse**: production of red-wine pigment, diffusible in the medium.	Coenocytic mycelium with abundant pyriform microconidia; free and arranged alternately throughout the mycelium.<br><br>**Microconidia size** ($\bar{X}$): 3.6 x 1.7µm.

**MX91**	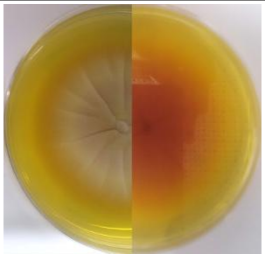	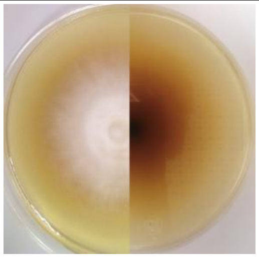	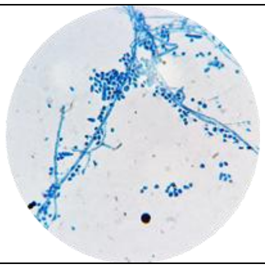
	**Obverse**: pale yellow colony, crater-shaped, with a velvety surface, with radial growth and a limited and regular border.<br><br>**Reverse**: production of yellow-orange pigment, diffusible in the medium.	**Obverse**: white colony, with a velvety surface, with radial growth, limited and regular border.<br><br>**Reverse**: production of brown-yellow pigment, diffusible in the medium.	Coenocytic mycelium with abundant round microconidia; to a lesser extent pyriform; arranged freely.<br><br>**Microconidia size** (X̅): 3.1 x 1.7μm.
**MX94**	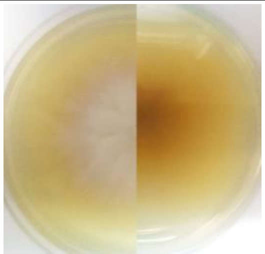	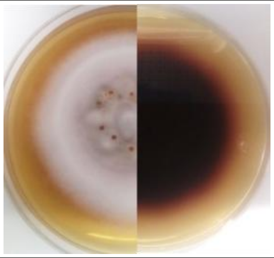	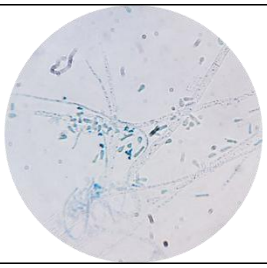
	**Obverse**: white colony, crateriform, with a velvety surface, with radial growth, limited and regular border.<br><br>**Reverse**: production of brown-yellow pigment, diffusible in the medium.	**Obverse**: white-pink colony, umbilicate center, hairy surface, radial growth, limited and regular border.<br><br>**Reverse**: production of red-wine pigment, diffusible in the medium.	Coenocytic mycelium with pyriform microconidia; free and arranged throughout the mycelium.<br><br>**Microconidia size** (X̅): 3.9 x 1.6μm.
**MX100**	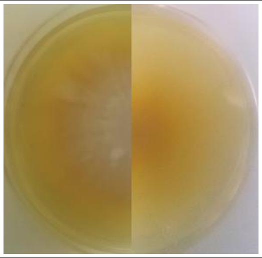	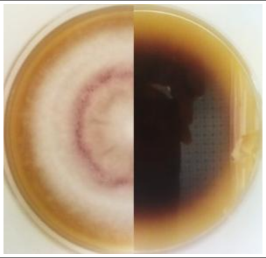	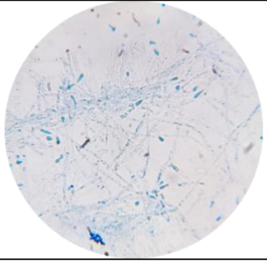
	**Obverse**: pale yellow colony, crater-shaped, with velvety surface, with radial growth, limited and regular border.<br><br>**Reverse**: production of brown-yellow pigment.	**Obverse**: colony white-pink, crateriform, with a cottony surface, with radial growth, limited and regular border.<br><br>**Reverse**: production of red-wine pigment, diffusible in the medium.	Septate mycelium with abundant pyriform microconidia; free and arranged throughout the mycelium.<br><br>**Microconidia size** (X̅): 4.0 x 1.8μm.

**MX122**	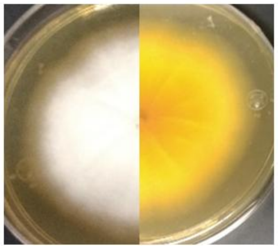	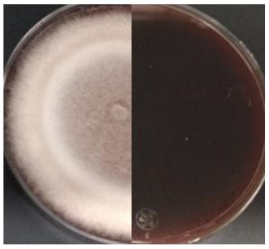	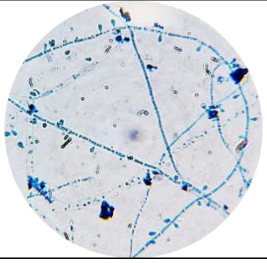
	**Obverse**: white colony, crateriform, with a velvety surface, with radial growth and a limited and regular border.<br><br>**Reverse**: production of yellow pigment, not diffusible in the medium.	**Obverse**: white-pink colony, with a hairy surface, with radial growth, limited and regular border.<br><br>**Reverse**: production of red-wine pigment, diffusible in the medium.	Coenocytic mycelium with few pyriform microconidia; arranged throughout the mycelium.<br><br>**Microconidia size** (X̅): 3.2 x 2.0µm.
**MX127**	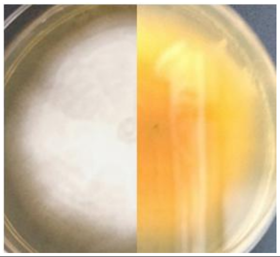	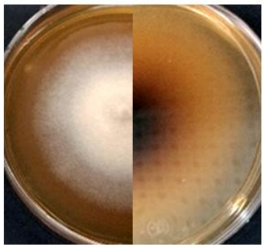	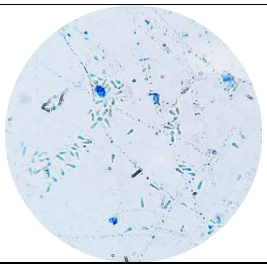
	**Obverse**: white colony, crateriform, with a velvety surface, with radial growth and a limited and regular border.<br><br>**Reverse**: production of yellow-orange pigment, not diffusible in the medium.	**Obverse**: white-pink colony, with a hairy surface, with radial growth, limited and regular border.<br><br>**Reverse**: production of brown-yellow pigment, diffusible in the medium.	Coenocytic mycelium with abundant pyriform microconidia; free and arranged throughout the mycelium.<br><br>**Microconidia size** (X̅): 3.1 x 1.7µm.
Trichophyton mentagrophytes			
**DO28**	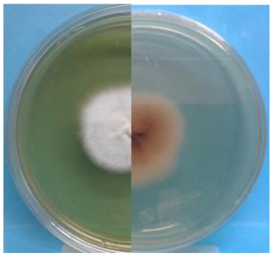	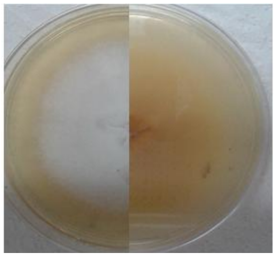	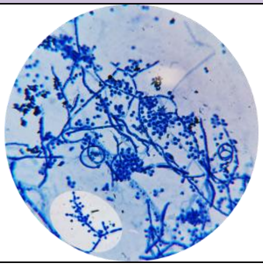
	**Obverse**: white colony, crateriform, with a powdery surface, with radial growth and a	**Obverse**: white colony, crateriform, with a powdery surface, with radial growth and a	Septate mycelium, formation of tendrils and abundant round microconidia, to a

	limited and regular border.<br><br>**Reverse**: production of brown pigment, not diffusible in the medium.	limited and regular border.<br><br>**Reverse**: pigmentation absent.	lesser extent pyriform, free; arranged along the mycelium or in the form of a "Lorraine cross".<br><br>**Microconidia size** ($\bar{X}$): 1.9 x 1.5μm.
**GT07**	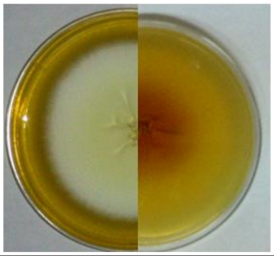	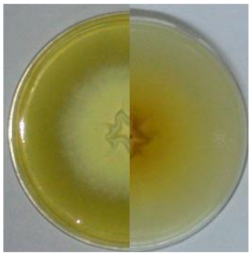	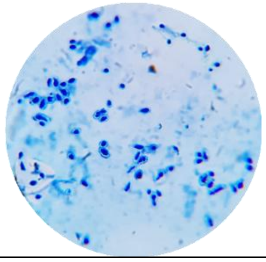
	**Obverse**: yellow-white colony, crateriform, with a hairy surface, with radial growth, limited and regular border.<br><br>**Reverse**: production of orange pigment, not diffusible in the medium.	**Obverse**: pale yellow colony, powdery surface with formation of folds in the center, limited and regular border.<br><br>**Reverse**: production of yellow pigment, not diffusible in the medium.	Coenocytic mycelium with abundant free round and pyriform microconidia.<br><br>**Microconidia size** ($\bar{X}$): 3.0 x 2.3μm.
**GT08**	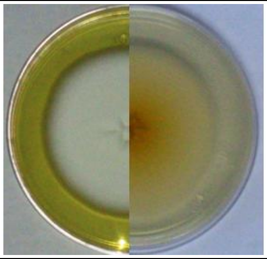	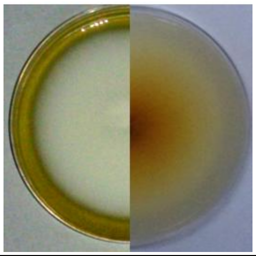	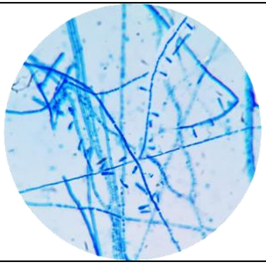
	**Obverse**: white colony, crateriform, cottony surface, with radial growth, limited and regular border.<br><br>**Reverse**: slight production of yellow-orange pigment, not diffusible in the medium.	**Obverse**: white colony, cottony surface, with radial growth, limited and regular border.<br><br>**Reverse**: production of yellow-orange pigment, not diffusible in the medium.	Septate mycelium with few pyriform microconidia; arranged throughout the mycelium.<br><br>**Microconidia size** ($\bar{X}$): 3.0 x 1.6μm.
**GT09**	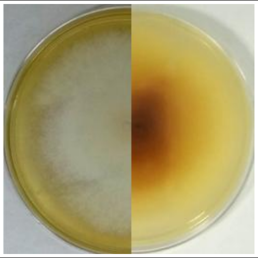	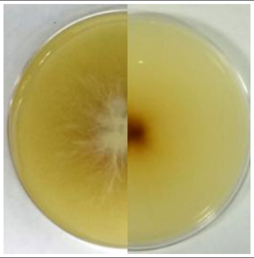	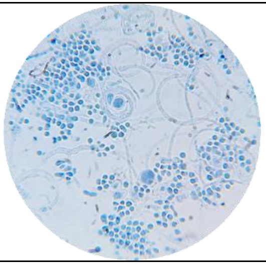
	**Obverse**: white colony, powdery surface, with radial growth,	**Obverse**: white colony, velvety surface, with radial growth,	Septate mycelium with formation of tendrils,

	irregular border.<br><br>**Reverse**: production of brown-yellow pigment, not diffusible in the medium.	irregular border.<br><br>**Reverse**: production of brown-yellow pigment, not diffusible in the medium.	abundant free round and pyriform microconidia.<br><br>**Microconidia size** (X̅): 3.0 x 2.4µm.
GT16	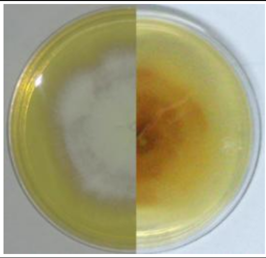	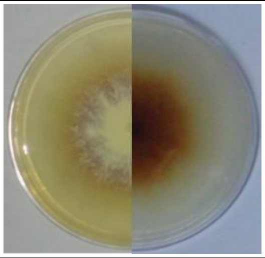	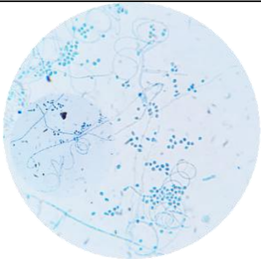
	**Obverse**: white colony, with a powdery surface, with radial growth, limited and regular border.<br><br>**Reverse**: slight production of light brown pigment, not diffusible in the medium.	**Obverse**: pale yellow colony, powdery surface, limited and irregular border.<br><br>**Reverse**: production of reddish-brown pigment, not diffusible in the medium.	Septate mycelium with formation of tendrils and free round and pyriform microconidia.<br><br>**Microconidia size** (X̅): 2.9 x 2.3µm.
GT18	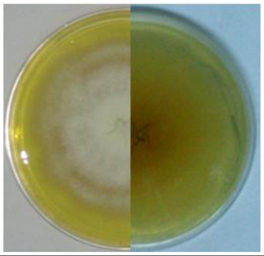	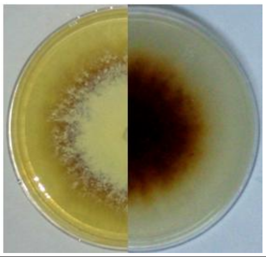	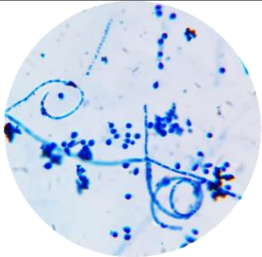
	**Obverse**: white colony, with a powdery surface, with radial growth, limited and regular border.<br><br>**Reverse**: production of light brown pigment, not diffusible in the medium.	**Obverse**: pale yellow colony, powdery surface, radial growth, limited and irregular border.<br><br>**Reverse**: production of reddish-brown pigment, not diffusible in the medium.	Septate mycelium with formation of tendrils and abundant free round and pyriform microconidia, arranged throughout the mycelium.<br><br>**Microconidia size** (X̅): 2.2 x 1.7µm.
GT22	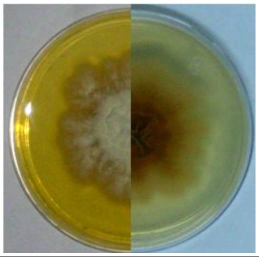	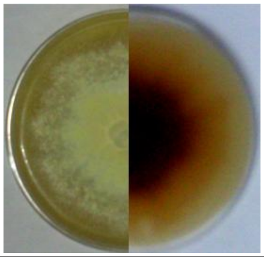	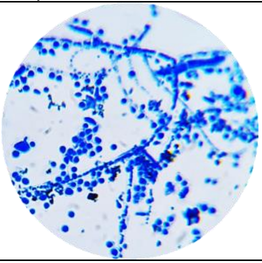
	**Obverse**: white colony, with a powdery surface, with radial	**Obverse**: pale yellow colony, crateriform, with a powdery	Coenocytic mycelium with abundant free round and

	growth, limited and irregular border.<br><br>**Reverse**: production of light brown pigment, not diffusible in the medium.	surface, with radial growth, limited and irregular border.<br><br>**Reverse**: production of reddish-brown pigment, diffusible in the medium.	pyriform microconidia; sparse, cigar-shaped, smooth-walled macroconidia with 2–3 locules.<br><br>**Microconidia size** ($\bar{X}$): 3.1 x 2.3µm.<br>**Macroconidia size** ($\bar{X}$): 17.8 x 3.4µm.
GT29	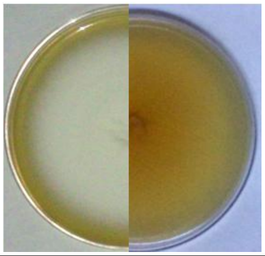	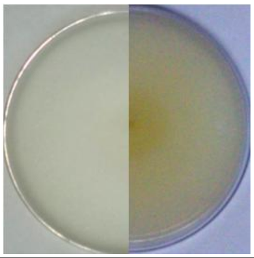	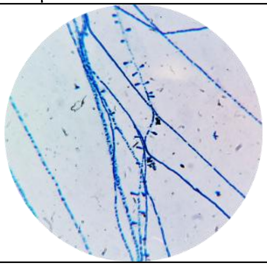
	**Obverse**: white colony, cottony surface, with radial growth, limited and regular border.<br><br>**Reverse**: pigmentation absent.	**Obverse**: white colony, cottony surface, with radial growth, limited and regular border.<br><br>**Reverse**: slight production of yellow pigment, not diffusible in the medium.	Coenocytic mycelium with abundant pyriform microconidia, arranged alternately along the mycelium.<br><br>**Microconidia size** ($\bar{X}$): 2.9 x 1.9µm.
GT30	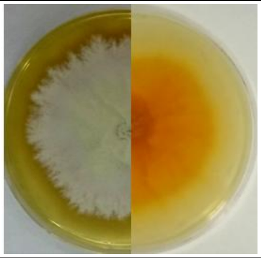	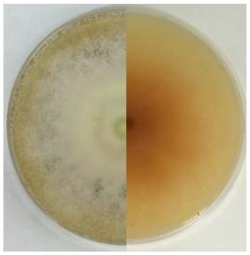	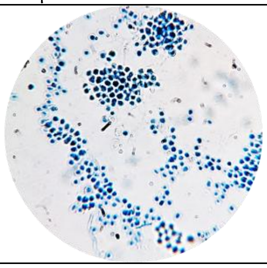
	**Obverse**: yellow-white colony, crater-shaped, with a powdery surface, with radial growth, limited and irregular border.<br><br>**Reverse**: production of yellow-orange pigment, diffusible in the medium.	**Obverse**: yellow-white colony, umbilicate, with a powdery surface, with radial growth, limited and regular border.<br><br>**Reverse**: production of light brown pigment, not diffusible in the medium.	Coenocytic mycelium with abundant free, round microconidia; and to a lesser extent pyriform.<br><br>**Microconidia size** ($\bar{X}$): 2.9 x 2.8µm.
GT39	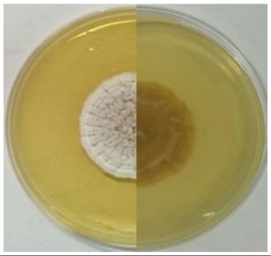	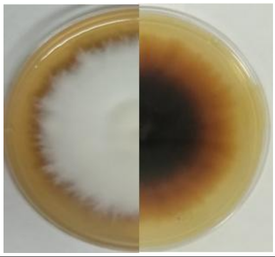	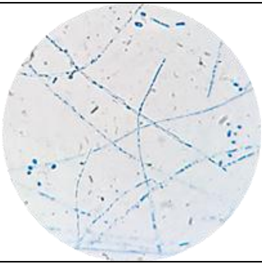
	**Obverse**: white colony,	**Obverse**: pale yellow-white	Septate mycelium with

	cerebriform, with a velvety surface, with radial growth, limited and regular border.<br><br>**Reverse**: pigmentation absent.	colony, umbilicate, with a floccose surface, with radial growth, limited and regular border.<br><br>**Reverse**: production of reddish-brown pigment, diffusible in the medium.	abundant free, round microconidia; and to a lesser extent pyriform.<br><br>**Microconidia size** ($\bar{X}$): 2.6 x 2.3µm.
GT40	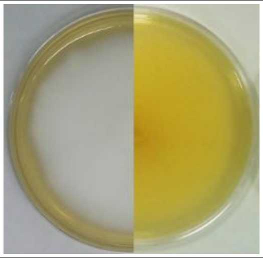	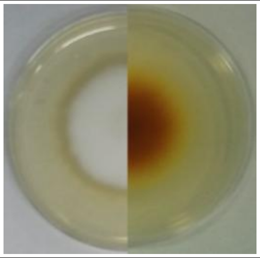	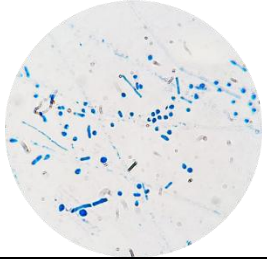
	**Obverse**: white colony, with radial growth, cottony surface, limited and regular border.<br><br>**Reverse**: pigmentation absent.	**Obverse**: white colony, cottony surface, with radial growth, limited and regular border.<br><br>**Reverse**: production of brown pigment, not diffusible in the medium.	Septate mycelium with abundant free round and pyriform microconidia; little presence of cigar-shaped macroconidia, smooth-walled with 2 to 5 locules.<br><br>**Microconidia size** ($\bar{X}$): 2.5 x 2.2µm.<br>**Macroconidia size** ($\bar{X}$): 28.4 x 4.3µm.
GT41	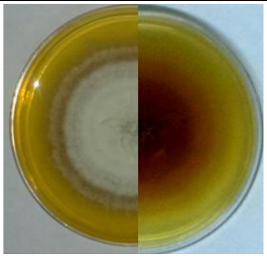	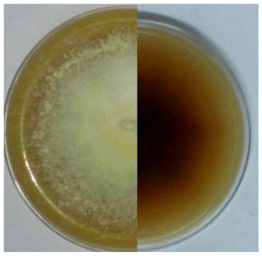	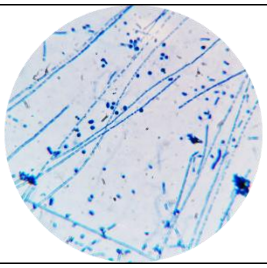
	**Obverse**: white colony, with a powdery surface, with radial growth, limited and regular border.<br><br>**Reverse**: production of brown pigment, not diffusible in the medium.	**Obverse**: pale yellow colony, powdery surface, radial growth, limited and irregular border.<br><br>**Reverse**: production of reddish-brown pigment, diffusible in the medium.	Septate mycelium with abundant free round and pyriform microconidia.<br><br>**Microconidia size** ($\bar{X}$): 2.3 x 2.1µm.

GT42	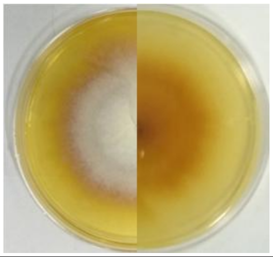	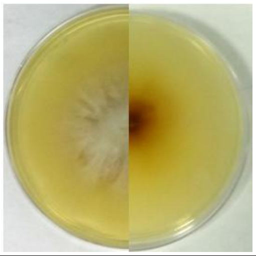	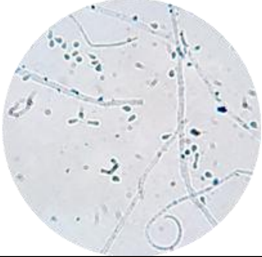
	**Obverse**: white colony, with a powdery surface, with radial growth, limited and regular border.<br><br>**Reverse**: production of brown-yellow pigment, not diffusible in the medium.	**Obverse**: white colony, with a velvety surface, with radial growth, limited and irregular border.<br><br>**Reverse**: slight production of brown-yellow pigment, not diffusible in the medium.	Septate mycelium with formation of tendrils and abundant round and pyriform microconidia; free and arranged throughout the mycelium.<br><br>**Microconidia size** ($\bar{X}$): 2.6 x 2.3μm.
GT43	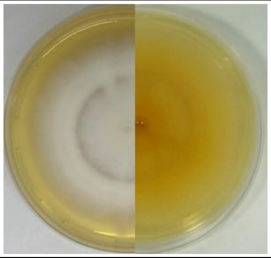	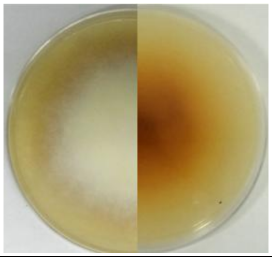	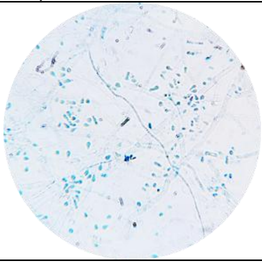
	**Obverse**: white colony, with a powdery surface, with radial growth, limited and regular border.<br><br>**Reverse**: slight production of brown-yellow pigment, not diffusible in the medium.	**Obverse**: pale yellow-white colony, cottony surface, with radial growth, limited and regular border.<br><br>**Reverse**: slight production of brown-yellow pigment, diffusible in the medium.	Septate mycelium with formation of tendrils and abundant round and pyriform microconidia; free and arranged throughout the mycelium.<br><br>**Microconidia size** ($\bar{X}$): 3.3 x 2.0μm.
GT45	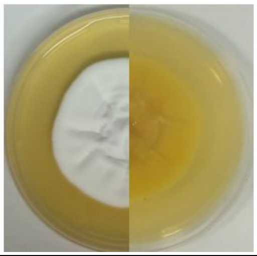	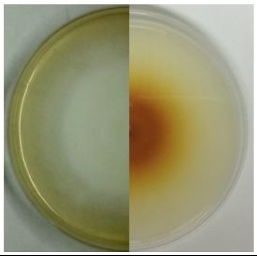	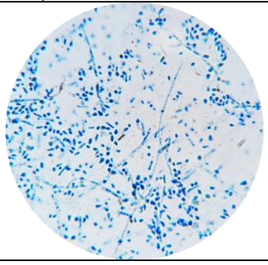
	**Obverse**: white colony, crateriform, with a powdery surface, with radial growth, limited and regular border.<br><br>**Reverse**: pigmentation absent.	**Obverse**: pale yellow-white colony, with a powdery surface, with radial growth, limited and regular border.<br><br>**Reverse**: production of yellow-orange pigment, not diffusible in the medium.	Septate mycelium with abundant free round and pyriform microconidia.<br><br>**Microconidia size** ($\bar{X}$): 2.9 x 2.1μm.

GT51	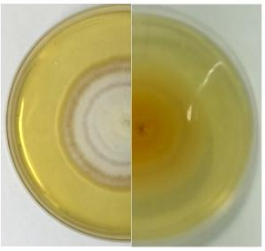	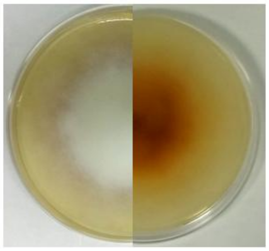	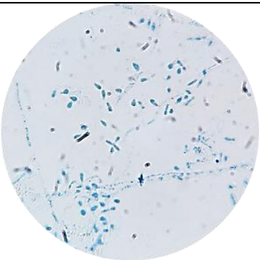
	**Obverse**: white colony, with a powdery surface, with radial growth, limited and regular border.<br><br>**Reverse**: slight production of brown-yellow pigment, not diffusible in the medium.	**Obverse**: white colony, cottony surface, with radial growth, limited and regular border.<br><br>**Reverse**: production of brown-yellow pigment, not diffusible in the medium.	Coenocytic mycelium with abundant free round and pyriform microconidia.<br><br>**Microconidia size** ($\bar{X}$): 2.3 x 1.7μm.
GT54	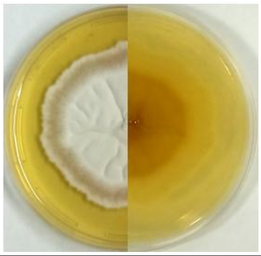	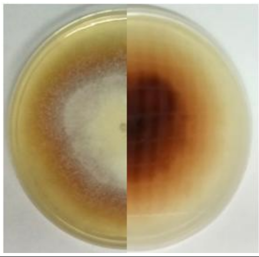	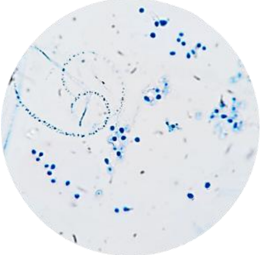
Descripción	**Obverse**: white colony, crateriform, with a powdery surface, with radial growth, limited and irregular border.<br><br>**Reverse**: pigmentation absent.	**Obverse**: pale yellow-white colony, with a powdery surface, with radial growth, limited and regular border.<br><br>**Reverse**: production of reddish-brown pigment, diffusible in the medium.	Septate mycelium with formation of tendrils and abundant free round and pyriform microconidia.<br><br>**Microconidia size** ($\bar{X}$): 3.5 x 2.5μm.
GT57	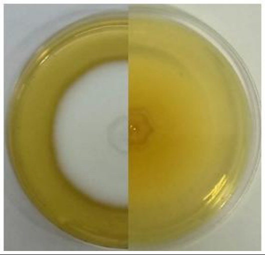	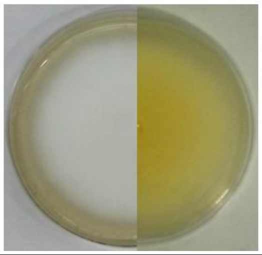	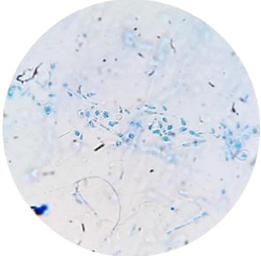
	**Obverse**: white colony, cottony surface, umbilicate, with radial growth, limited and regular border.<br><br>**Reverse**: pigmentation absent.	**Obverse**: white colony, cottony surface, with radial growth, limited and regular border.<br><br>**Reverse**: slight production of yellow pigment, not diffusible in the medium.	Septate mycelium with formation of tendrils and abundant free round and pyriform microconidia.<br><br>**Microconidia size** ($\bar{X}$): 3.5 x 2.0μm.

GT58	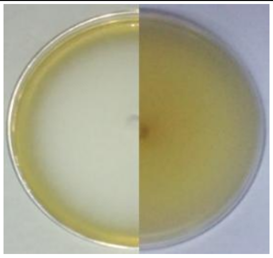	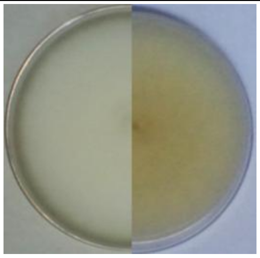	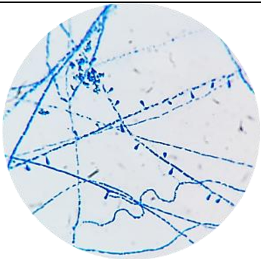
	**Obverse**: white colony, cottony surface, with radial growth, limited and regular border.<br><br>**Reverse**: pigmentation absent.	**Obverse**: white colony, cottony surface, with radial growth, limited and regular border.<br><br>**Reverse**: pigmentation absent.	Septate mycelium with abundant round and pyriform microconidia, free, arranged along the mycelium.<br><br>**Microconidia size** ($\bar{X}$): 3.0 x 1.6µm.
GT59	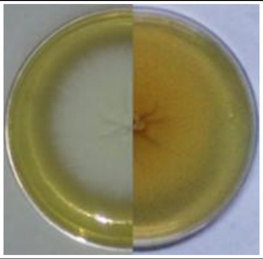	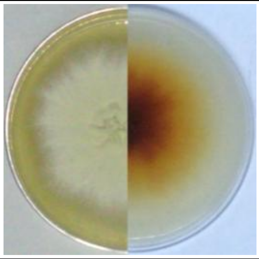	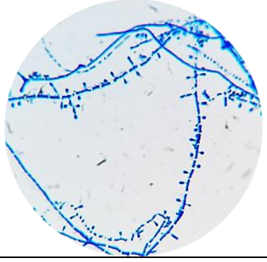
	**Obverse**: pale yellow-white colony, with a velvety surface, with radial growth, limited and regular border.<br><br>**Reverse**: pigmentation absent.	**Obverse**: pale yellow colony, crateriform, with a powdery surface, with radial growth, limited and irregular border.<br><br>**Reverse**: production of brown-yellow pigment, not diffusible in the medium.	Septate mycelium with abundant pyriform microconidia, arranged along the mycelium and to a lesser extent forming a "Lorraine cross".<br><br>**Microconidia size** ($\bar{X}$): 2.4 x 1.1µm.
GT62	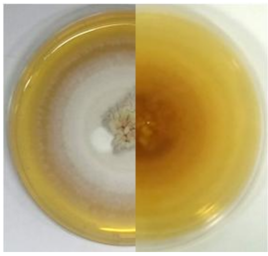	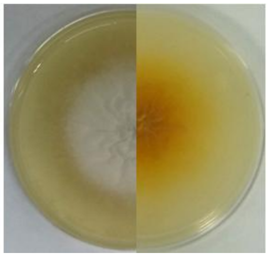	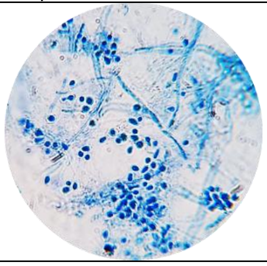
	**Obverse**: white colony, cerebriform, with a powdery surface, with radial growth, limited and regular border.<br><br>**Reverse**: slight production of light brown pigment, not diffusible in the medium.	**Obverse**: white colony, crateriform, with a powdery surface, with radial growth, limited and regular border.<br><br>**Reverse**: production of yellow-orange pigment, not diffusible in the medium.	Septate mycelium with abundant free, round microconidia; and to a lesser extent pyriform.<br><br>**Microconidia size** ($\bar{X}$): 2.5 x 2.2µm.

GT63	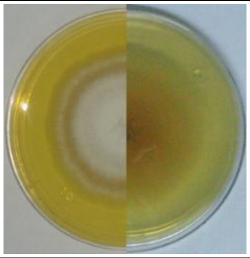	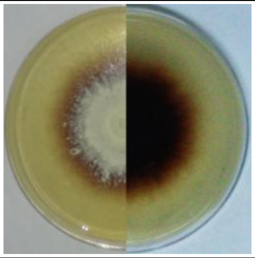	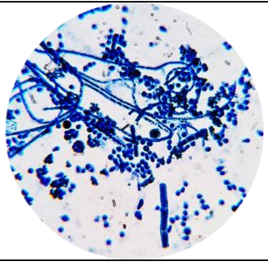
	**Obverse**: white colonies, with a powdery surface, with radial growth, limited and regular border.<br><br>**Reverse**: pigmentation absent.	**Obverse**: pale yellow colonies, with a powdery surface, with radial growth, limited and irregular border.<br><br>**Reverse**: production of reddish-brown pigment, not diffusible in the medium.	Septate mycelium with formation of tendrils and abundant free, round and pyriform microconidia; scant presence of cigar-shaped macroconidia, smooth-walled with 2 to 3 locules.<br><br>**Microconidia size** ($\bar{X}$): 2.6 x 1.9µm.<br>**Macroconidia size** ($\bar{X}$):: 14.0 x 2.6µm.
GT65	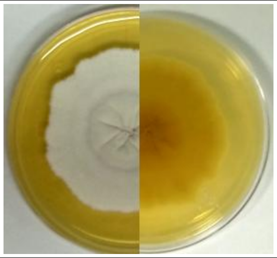	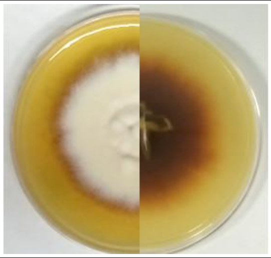	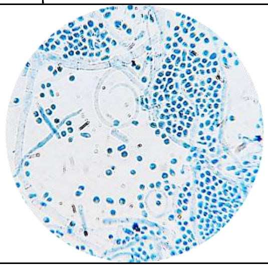
	**Obverse**: white colony, crateriform, with a powdery surface, with radial growth, limited and irregular border.<br><br>**Reverse**: pigmentation absent.	**Obverse**: white colony, with a floccose surface, with radial growth, limited and regular border.<br><br>**Reverse**: production of reddish-brown pigment, diffusible in the medium.	Septate mycelium with formation of tendrils and abundant free, pyriform microconidia.<br><br>**Microconidia size** ($\bar{X}$): 2.7 x 2.3µm.
HN02	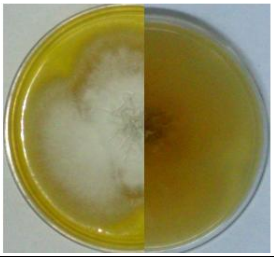	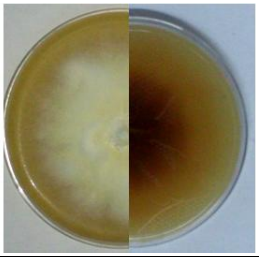	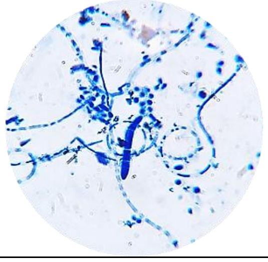
	**Obverse**: white colony, cerebriform, with a powdery surface, with radial growth,	**Obverse**: pale yellow colony, powdery surface, limited and irregular border.	Coenocytic mycelium with tendril formation and abundant free, round

	limited and irregular border.<br><br>**Reverse**: pigmentation absent.	**Reverse**: production of reddish-brown pigment, not diffusible in the medium.	microconidia; sparse, cigar-shaped, smooth-walled macroconidia with 2–3 locules.<br><br>**Microconidia size** ($\bar{X}$) 2.5 x 2.0µm.<br>**Macroconidia size** ($\bar{X}$): 17.9 x 3.8µm.
MX69	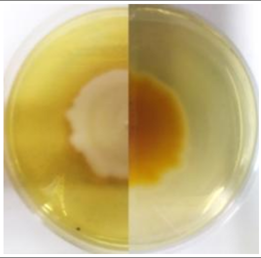	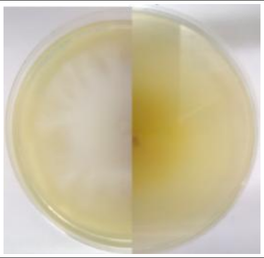	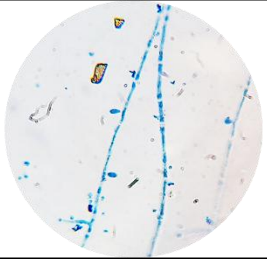
	**Obverse**: pale yellow-white colony, with a powdery surface, with radial growth, limited and regular border.<br><br>**Reverse**: production of orange pigment, not diffusible in the medium.	**Obverse**: white colony, with a powdery surface, with radial growth, limited and regular border.<br><br>**Reverse**: slight production of yellow pigment, not diffusible in the medium.	Septate mycelium with few pyriform microconidia, arranged along the mycelium.<br><br>**Microconidia size** ($\bar{X}$): 2.1 x 1.1µm.
MX79	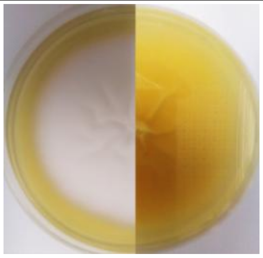	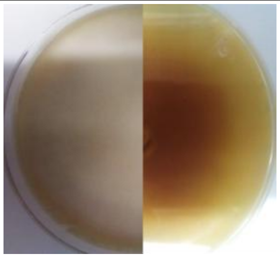	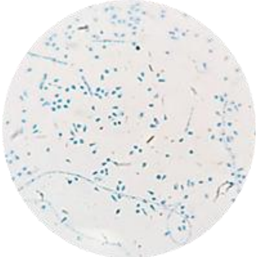
	**Obverse**: white colony, crateriform, with a powdery surface, with radial growth, limited and regular border.<br><br>**Reverse**: slight production of orange pigment, not diffusible in the medium.	**Obverse**: pale yellow colony, powdery surface, radial growth, limited and regular border.<br><br>**Reverse**: production of brown pigment, not diffusible in the medium.	Coenocytic mycelium with abundant round and pyriform microconidia; free and arranged throughout the mycelium; little presence of cigar-shaped macroconidia, smooth-walled with 2 locules.<br><br>**Microconidia size** ($\bar{X}$): 5.5 x 4.3µm.<br>**Macroconidia size** ($\bar{X}$): 14.9 x 2.2µm.

MX83	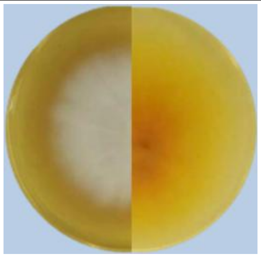	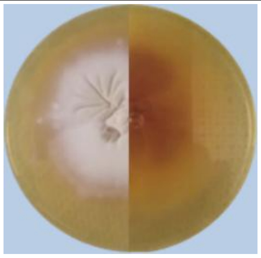	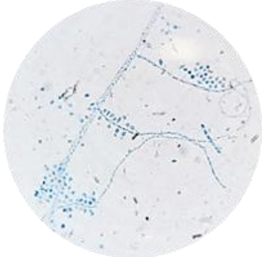
	**Obverse**: white colony, crateriform, with a powdery surface, with radial growth, limited and regular border.<br><br>**Reverse**: production of orange pigment, not diffusible in the medium.	**Obverse**: colony white, crateriform, powdery surface, with radial growth, limited and regular border.<br><br>**Reverse**: production of brown pigment, not diffusible in the medium.	Coenocytic mycelium with formation of tendrils and abundant free round and pyriform microconidia.<br><br>**Microconidia size** ($\bar{x}$): 2.8 x 2.1µm.
Trichophyton tonsurans			
MX55	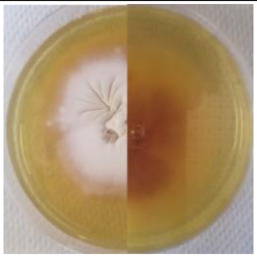	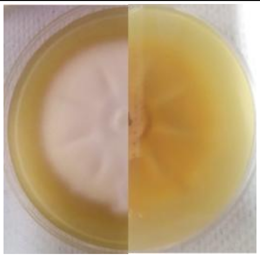	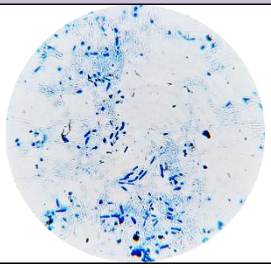
	**Obverse**: pale yellow to white colony, crater-shaped, with a powdery surface, with radial growth, limited and regular border.<br><br>**Reverse**: slight production of brown pigment, not diffusible in the médium.	**Obverse**: white colony, umbilicate, with a powdery surface, with radial growth, limited and regular border.<br><br>**Reverse**: pigmentation absent.	Septate mycelium with abundant pyriform microconidia, to a lesser extent round, free.<br><br>**Microconidia size** ($\bar{x}$): 3.9 x 2.1µm.
MX81	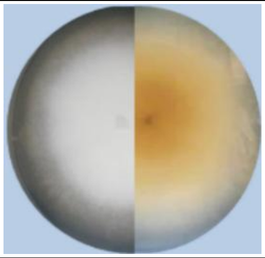	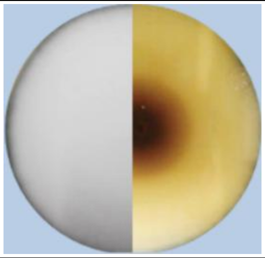	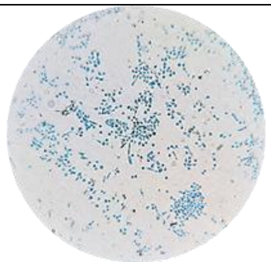
	**Obverse**: white colony, with a velvety surface, with radial growth, limited and regular border.<br><br>**Reverse**: slight production of brown-yellow pigment, not	**Obverse**: white colonies, cottony surface, with radial growth, limited and regular border.<br><br>**Reverse**: slight production of brown and yellow pigment, diffusible in the medium.	Coenocytic mycelium with abundant free round and pyriform microconidia.<br><br>**Microconidia size** ($\bar{x}$): 3.5 x 1.9µm.

	diffusible in the medium.		
MX82	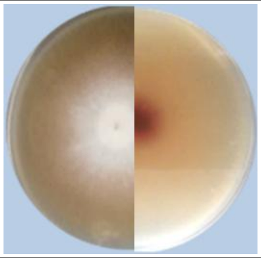	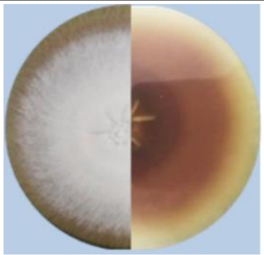	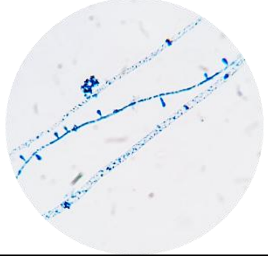
	**Obverse**: white colony, with a velvety surface, with radial growth, limited and regular border.<br><br>**Reverse**: slight production of brown pigment, not diffusible in the medium.	**Obverse**: white colony, crateriform, with a powdery surface, with radial growth, limited and regular border.<br><br>**Reverse**: dark brown pigment production.	Coenocytic mycelium with round and pyriform microconidia; arranged throughout the mycelium.<br><br>**Microconidia size** ($\bar{X}$): 3.6 x 2.2µm.
MX85	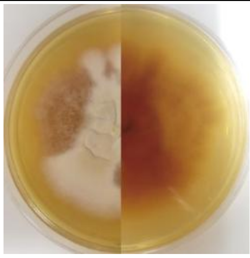	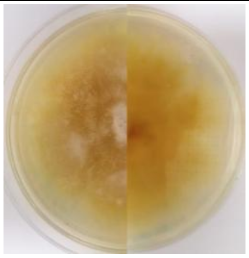	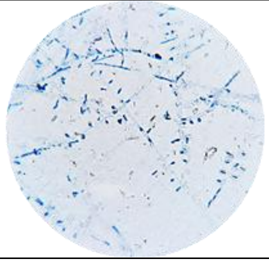
	**Obverse**: dim yellow to white colony, crateriform, with a powdery surface, with radial growth, limited and irregular border.<br><br>**Reverse**: slight production of brown pigment, not diffusible in the medium.	**Obverse**: colony yellow and white, powdery surface, irregular border.<br><br>**Reverse**: production of brown-yellow pigment, diffusible in the medium.	Septate mycelium with abundant round and pyriform microconidia; free and arranged throughout the mycelium.<br><br>**Microconidia size** ($\bar{X}$): 3.7 x 1.8µm.
MX86	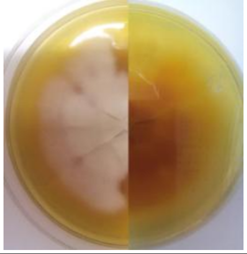	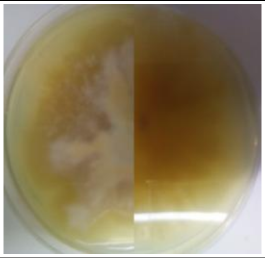	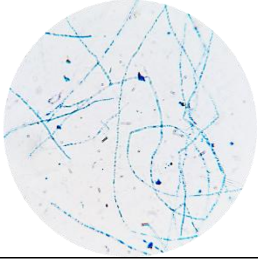
	**Obverse**: white colony, crateriform, with a powdery surface, with radial growth, limited and irregular border.<br><br>**Reverse**: production of brown-yellow pigment, diffusible in the	**Obverse**: yellow and white colony, with a powdery surface, with radial growth, limited and irregular border.<br><br>**Reverse**: production of brown-yellow pigment, diffusible in the	Coenocytic, sterile mycelium.

	medium.	medium.	
MX102	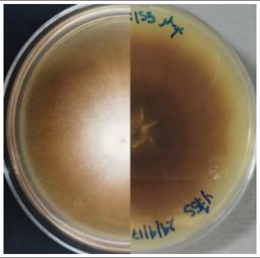	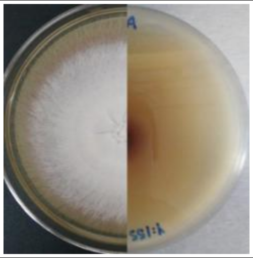	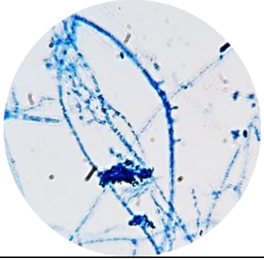
	**Obverse**: white colony, with a powdery surface, with radial growth, limited and regular border.<br><br>**Reverse**: production of brown pigment, diffusible in the medium.<br><br>.	**Obverse**: white colony, with a powdery surface, with radial growth, limited and regular border.<br><br>**Reverse**: production of brown-yellow pigment, diffusible in the medium.	Coenocytic mycelium with few pyriform microconidia, arranged along the mycelium.<br><br>**Microconidia size** (X̅): 3.8 x 1.8µm.
Microsporum gypseum			
GT02	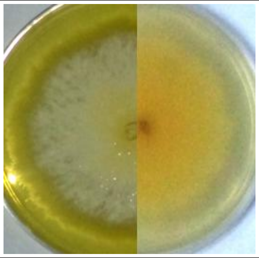	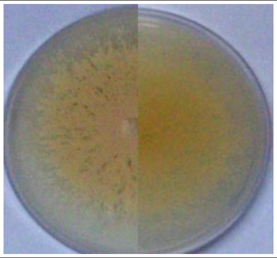	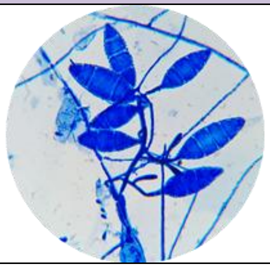
	**Obverse**: faint yellow to white colony, powdery surface, radial growth, limited and regular border.<br><br>**Reverse**: slight production of yellow pigment, not diffusible in the medium.	**Obverse**: ocher colony, with a powdery surface, with radial growth, limited and irregular border.<br><br>**Reverse**: pigmentation absent.	Septate mycelium with few spindle-shaped, thin-walled macroconidia; with 4 to 6 locules; few pyriform microconidia.<br><br>**Microconidia size** (X̅): 4.5 x 1.9µm.<br>**Macroconidia size** (X̅): 42.4 x 15.1µm.
GT03	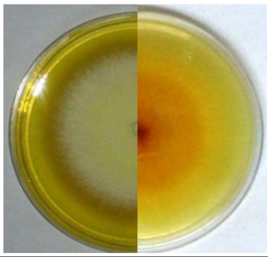	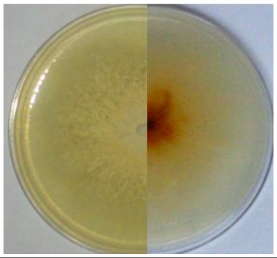	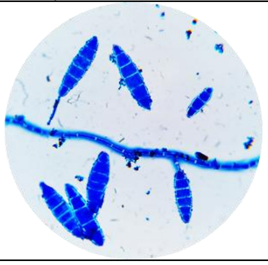
	**Obverse**: faint yellow to white colony, powdery surface, radial growth, limited and regular border.	**Obverse**: ocher colony, with a powdery surface, with radial growth, limited and irregular border.	Septate mycelium with abundant spindle-shaped, thin-walled macroconidia; with 5 to 6 locules.

	**Reverse**: production of yellow-orange pigment, diffusible in the medium.	**Reverse**: production of brown-yellow pigment, not diffusible in the medium.	**Macroconidia size** (X̄): 42.4 x 10.8µm.
GT04	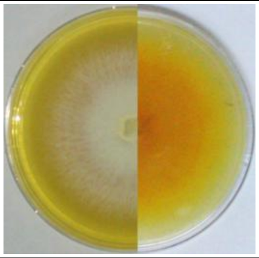	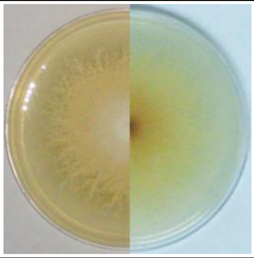	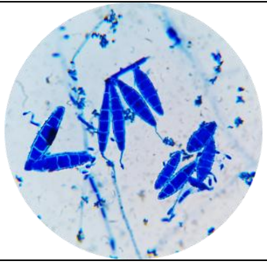
	**Obverse**: white colony, with a powdery surface, with radial growth, limited and regular border.<br><br>**Reverse**: production of yellow-orange pigment, diffusible in the medium.	**Obverse**: white colony, powdery surface, with radial growth, irregular border.<br><br>**Reverse**: slight production of brown pigment, not diffusible in the medium.	Septate mycelium with abundant spindle-shaped, thin-walled macroconidia; with 4 to 6 locules; abundant pyriform microconidia.<br><br>**Microconidia size** (X̄): 4.8 x 1.8µm.<br>**Macroconidia size** (X̄): 42.6 x 9.0µm.
GT10	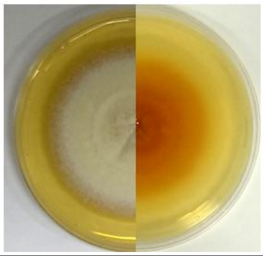	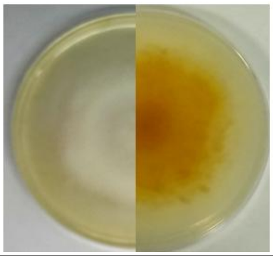	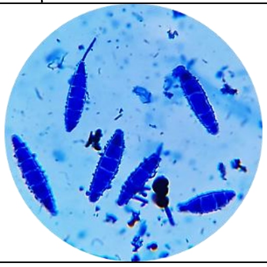
	**Obverse**: pale yellow-white colony, with a powdery surface, with radial growth, limited and regular border.<br><br>**Reverse**: production of brown-yellow pigment, not diffusible in the medium.	**Obverse**: white colony, umbilicate, with a cottony surface, with radial growth, limited and regular border.<br><br>**Reverse**: production of brown-yellow pigment, not diffusible in the medium.	Septate mycelium, with abundant spindle-shaped, thin-walled macroconidia; with 5 to 6 locules.<br><br>**Macroconidia size** (X̄): 41.1 x 9.6µm.
GT11	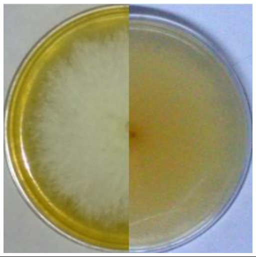	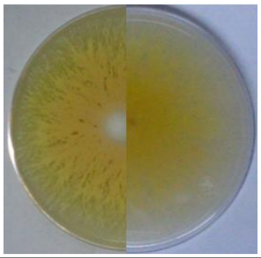	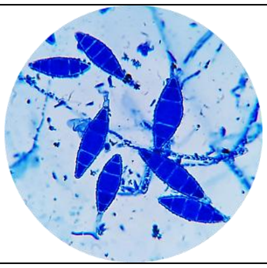
	**Obverse**: pale yellow-white colony, with a powdery surface, with radial growth, limited and regular border.	**Obverse**: white colony, powdery surface, with radial growth, irregular border.	Septate mycelium with few spindle-shaped, thin-walled macroconidia; with 4 to 6 locules; pyriform

	**Reverse**: pigmentation absent.	**Reverse**: pigmentation absent.	microconidia.<br><br>**Microconidia size** ($\bar{X}$): 4.2 x 1.6µm.<br>**Macroconidia size** ($\bar{X}$): 43.3 x 15.2µm.
GT13	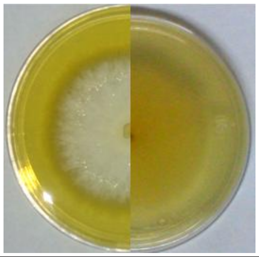	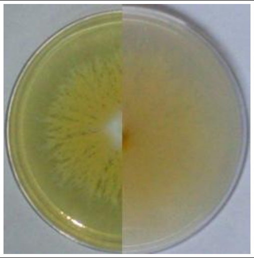	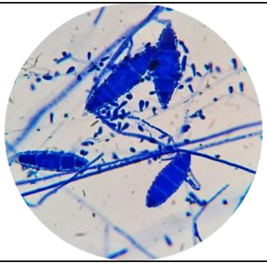
	**Obverse**: pale yellow-white colony, with a powdery surface, with radial growth, limited and regular border.<br><br>**Reverse**: pigmentation absent.	**Obverse**: ocher colony, with a powdery surface, with radial growth, limited and irregular border.<br><br>**Reverse**: pigmentation absent.	Septate mycelium with abundant spindle-shaped, thin-walled macroconidia; with 4 to 6 locules; pyriform microconidia.<br><br>**Microconidia size** ($\bar{X}$): 5.8 x 2.3µm.<br>**Macroconidia size** ($\bar{X}$): 37.7 x 10.0µm.
GT15	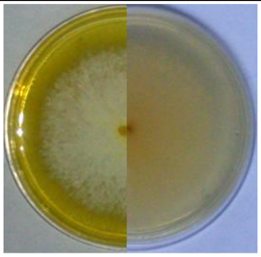	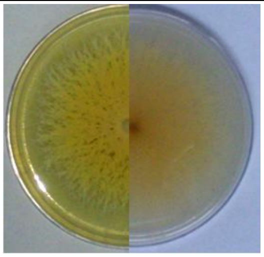	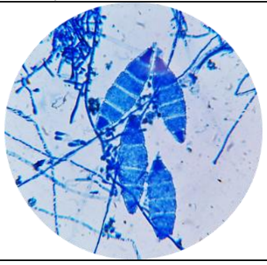
	**Obverse**: pale yellow to white colony, powdery surface, radial growth, limited and regular border.<br><br>**Reverse**: pigmentation absent.	**Obverse**: ocher colony, with a powdery surface, with radial growth, irregular border.<br><br>**Reverse**: pigmentation absent.	Septate mycelium with abundant spindle-shaped, thin-walled macroconidia; with 4 to 6 locules; few pyriform microconidia.<br><br>**Microconidia size** ($\bar{X}$): 3.7 x 1.9µm.<br>**Macroconidia size** ($\bar{X}$): 43.9 x 16.0µm.

GT17	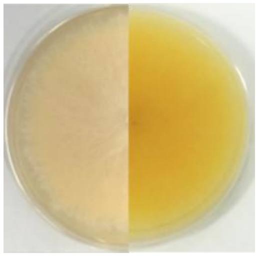	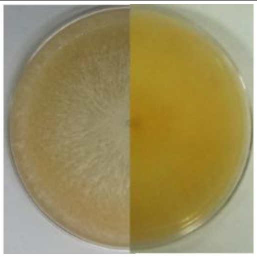	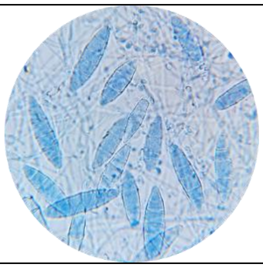
	**Obverse**: ochre-colored colony, with a powdery surface, with radial growth, regular border.<br><br>**Reverse**: slight production of yellow pigment, diffusible in the medium.	**Obverse**: white colony, powdery surface, with radial growth, irregular border.<br><br>**Reverse**: pigmentation absent.	Septate mycelium with abundant spindle-shaped, thin-walled macroconidia; with 5-6 locules; few pyriform microconidia.<br><br>**Microconidia size** (X̅): 5.3 x 2.6µm.<br>**Macroconidia size** (X̅): 44.8 x 11.1µm.
GT24	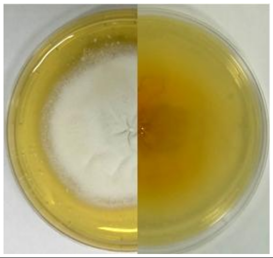	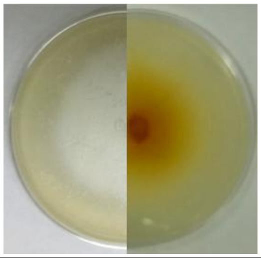	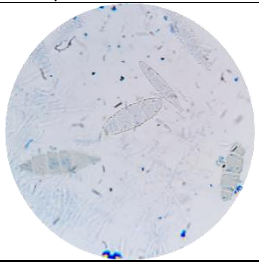
	**Obverse**: pale yellow-white colony, crater-shaped, with a powdery surface, with radial growth, limited and regular border.<br><br>**Reverse**: slight production of brown-yellow pigment, not diffusible in the medium.	**Obverse**: white colony, cottony surface, with radial growth, limited and regular border.<br><br>**Reverse**: production of brown-yellow pigment, not diffusible in the médium.	Septate mycelium with abundant spindle-shaped, thin-walled macroconidia; with 5-6 locules; abundant pyriform microconidia.<br><br>**Microconidia size** (X̅): 4.1 x 1.9µm.<br>Macroconidia size (X̅): 38.8 x 9.1µm.
GT26	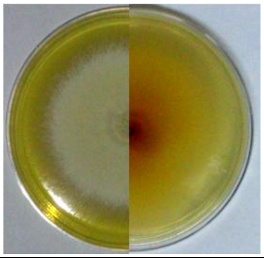	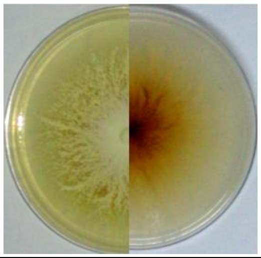	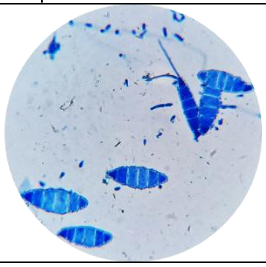
	**Obverse**: pale yellow to white colony, umbilicated, powdery surface, radial growth, limited and regular border.<br><br>**Reverse**: production of yellow-	**Obverse**: ocher colony, with a powdery surface, with radial growth, limited and irregular border.<br><br>**Reverse**: production of brown-	Septate mycelium with abundant spindle-shaped, thin-walled macroconidia; with 5-6 locules; few pyriform microconidia.

	orange pigment, diffusible in the medium.	yellow pigment, not diffusible in the medium.	**Microconidia size** (X̄): 3.6 x 1.6µm.<br>**Macroconidia size** (X̄): 39.8 x 10.0µm.
GT28	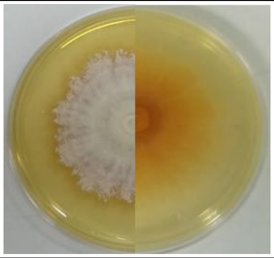	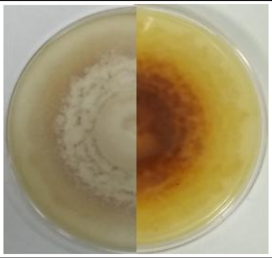	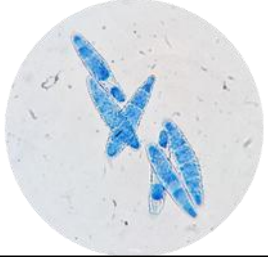
	**Obverse**: white colony, umbilicated, with a powdery surface, with radial growth, limited and irregular border.<br><br>**Reverse**: slight production of light brown pigment, not diffusible in the medium.	**Obverse**: ocher colony, with a powdery surface, with radial growth, limited and irregular border.<br><br>**Reverse**: production of brown to yellow pigment, diffusible in the medium.	Septate mycelium with abundant macroconidia fusiform, thin-walled; with 5 to 6 locules.<br><br>**Macroconidia size** (X̄): 47.1 x 9.6µm.
GT31	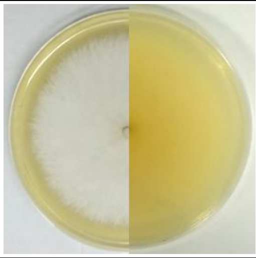	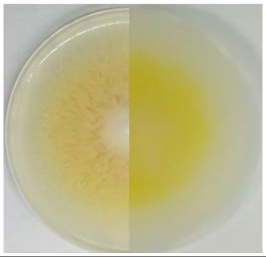	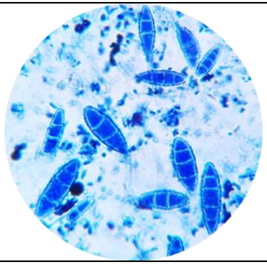
	**Obverse**: white colony, with a powdery surface, with radial growth, limited and regular border.<br><br>**Reverse**: pigmentation absent.	**Obverse**: ocher colony, with a powdery surface, with radial growth, limited and irregular border.<br><br>**Reverse**: production of yellow pigment, not diffusible in the medium.<br><br>.	Septate mycelium with abundant spindle-shaped macroconidia, thin-walled, with 3 to 6 locules; abundant pyriform microconidia.<br><br>**Microconidia size** (X̄): 3.7 x 2.0µm.<br>**Macroconidia size** (X̄): 44.6 x 11.8µm.
GT33	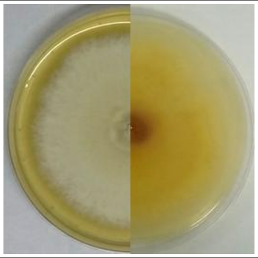	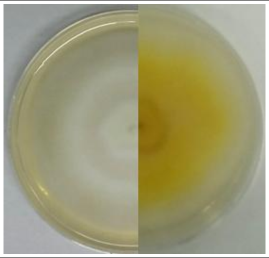	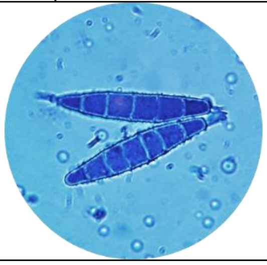
	**Obverse**: pale yellow to white colony, powdery surface, radial	**Obverse**: white colony, cottony surface, with radial growth,	Septate mycelium with abundant spindle-shaped

	growth, limited and regular border.<br><br>**Reverse**: production of brown-yellow pigment, diffusible in the medium.	limited and regular border.<br><br>**Reverse**: production of yellow pigment, not diffusible in the medium.	macroconidia, thin-walled, with 5 to 6 locules.<br><br>**Macroconidia size** ($\bar{X}$): 42.7 x 10.1µm.
GT48	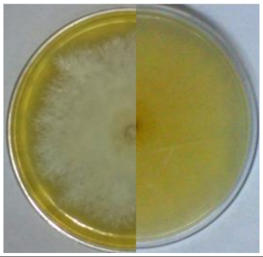	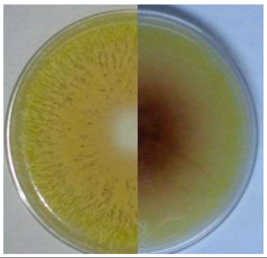	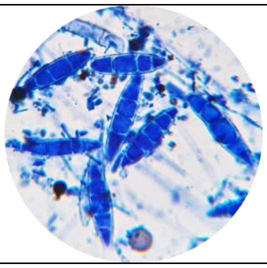
	**Obverse**: pale yellow-white colony, with a powdery surface, with radial growth, limited and regular border.<br><br>**Reverse**: pigmentation absent.	**Obverse**: ocher colony, with a powdery surface, with radial growth, irregular border.<br><br>**Reverse**: production of reddish-brown pigment, not diffusible in the medium.	Septate mycelium with abundant spindle-shaped, thin-walled macroconidia; with 4 to 6 locules; abundant pyriform microconidia.<br><br>**Microconidia size** ($\bar{X}$): 4.5 x 2.2µm.<br>**Macroconidia size** ($\bar{X}$): 43.5 x 10.9µm.
GT49	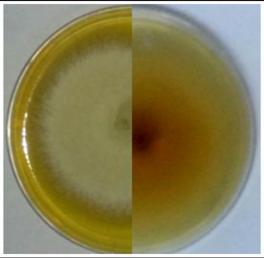	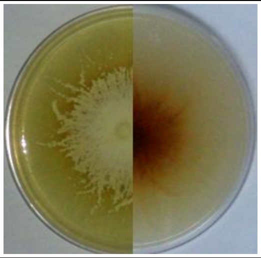	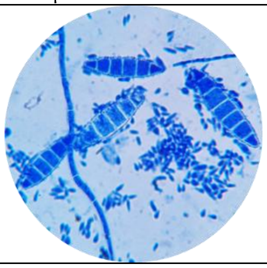
	**Obverse**: pale yellow to white colony, powdery surface, radial growth, limited and regular border.<br><br>**Reverse**: production of brown-yellow pigment, not diffusible in the medium.	**Obverse**: ochre-colored colony, with a powdery surface, with radial growth, limited and irregular border.<br><br>**Reverse**: production of brown-yellow pigment, not diffusible in the medium, presence of brown ramifications.	Septate mycelium with abundant spindle-shaped macroconidia, thin-walled, with 4 to 6 locules; abundant pyriform microconidia.<br><br>**Microconidia size** ($\bar{X}$): 4.9 x 1.7µm.<br>**Macroconidia size** ($\bar{X}$): 39.9 x 9.6µm.

GT52	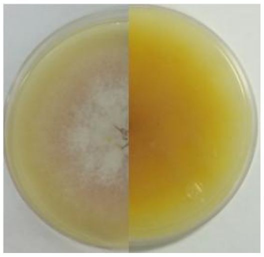	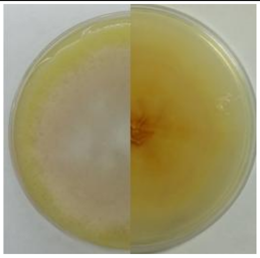	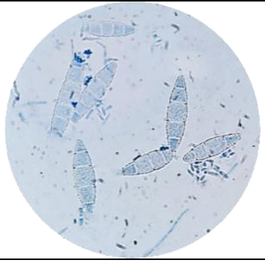
	**Obverse**: white colony, crateriform, with a powdery surface, with radial growth, limited and regular border.<br><br>**Reverse**: slight production of orange-yellow pigment, not diffusible in the medium.	**Obverse**: ocher-white colony, with a powdery surface, with radial growth, limited and regular border.<br><br>**Reverse**: production of yellow pigment, diffusible in the medium.	Septate mycelium with abundant spindle-shaped macroconidia, thin-walled, with 6 locules; few pyriform microconidia.<br><br>**Microconidia size** ($\bar{X}$): 4.2 x 2.1μm.<br>**Macroconidia size** ($\bar{X}$): 46.0 x 9.6μm.
GT55	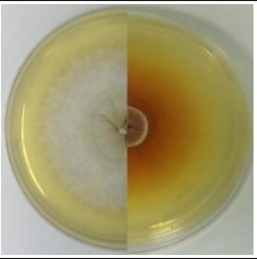	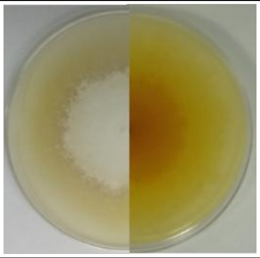	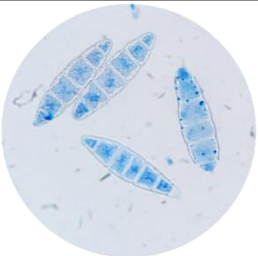
	**Obverse**: pale yellow colony, umbilicate, with powdery surface, with radial growth, limited and regular border.<br><br>**Reverse**: production of yellow-orange pigment, diffusible in the medium.	**Obverse**: white colony, with a powdery surface, with radial growth, limited and regular borders.<br><br>**Reverse**: production of orange-yellow pigment, diffusible in the medium.	Septate mycelium with abundant spindle-shaped macroconidia, thin-walled, with 5 to 6 locules.<br><br>**Macroconidia size** ($\bar{X}$): 45.8 x 10.6μm.
GT68	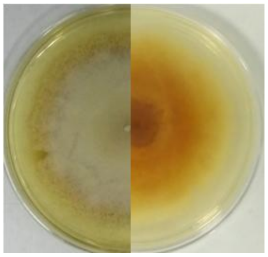	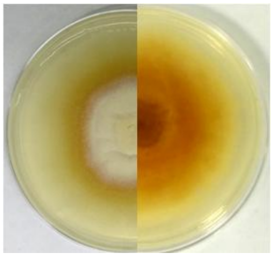	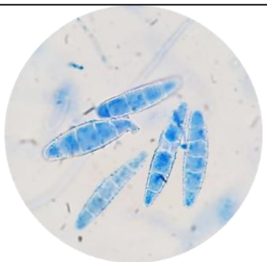
	**Obverse**: pale yellow-white colony, with a powdery surface, with radial growth, limited and irregular border.	**Obverse**: pale yellow-white colony, umbilicated, with a powdery surface, with radial growth, limited and regular border.	Septate mycelium with abundant thin-walled, spindle-shaped macroconidia with 6 locules.

	**Reverse**: production of brown-yellow pigment, not diffusible in the medium.	**Reverse**: production of brown-yellow pigment, diffusible in the medium.	**Macroconidia size** ($\bar{X}$): 43.6 x 11.8μm.
GT71	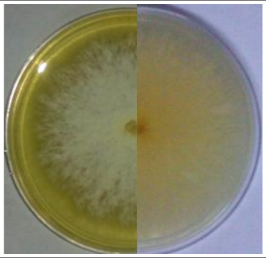	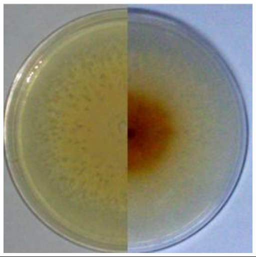	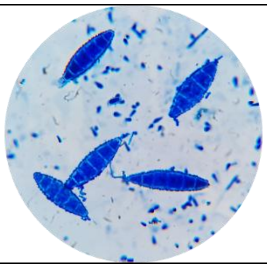
	**Obverse**: pale yellow colony, powdery surface, radiating in growth, regular border.<br><br>**Reverse**: pigmentation absent.	**Obverse**: ocher colony, with a powdery surface, with radial growth, limited and irregular border.<br><br>**Reverse**: production of brown-yellow pigment, not diffusible in the medium.	Septate mycelium with abundant spindle-shaped macroconidia, thin-walled, with 4 to 6 locules; few pyriform microconidia.<br><br>**Microconidia size** ($\bar{X}$): 3.5 x 1.9μm.<br>**Macroconidia size** ($\bar{X}$): 42.2 x 13.0μm.
GT75	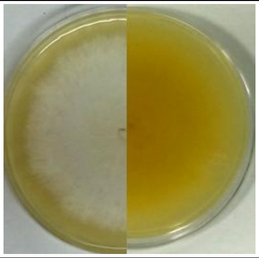	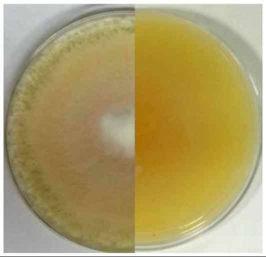	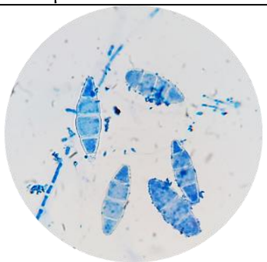
	**Obverse**: white colony, with a powdery surface, with radial growth, limited and regular border.<br><br>**Reverse**: production of yellow pigment, diffusible in the medium.	**Obverse**: ocher colony, with a powdery surface, with radial growth, irregular border.<br><br>**Reverse**: production of yellow pigment, diffusible in the medium.	Septate mycelium with abundant spindle-shaped macroconidia, thin-walled, with 3 to 4 locules; abundant pyriform microconidia.<br><br>**Microconidia size** ($\bar{X}$): 4.8 x 2.1μm.<br>**Macroconidia size** ($\bar{X}$): 34.7 x 11.1μm.
HN01	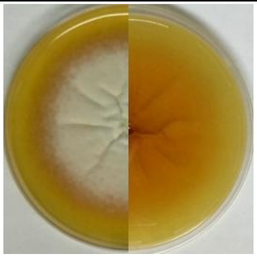	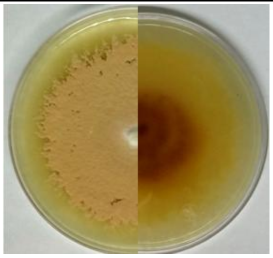	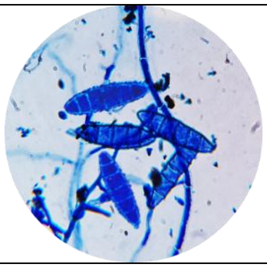

	**Obverse**: white colony, crateriform, with a powdery surface, with radial growth, limited and regular border.<br><br>**Reverse**: production of brown-yellow pigment, diffusible in the medium.	**Obverse**: ocher colony, with a powdery surface, with radial growth, limited and irregular border.<br><br>**Reverse**: production of brown-yellow pigment, diffusible in the medium.	Septate mycelium with abundant spindle-shaped macroconidia, thin-walled, with 5 to 6 locules; abundant pyriform microconidia.<br><br>**Microconidia size** (X̅): 3.6 x 2.0µm.<br>**Macroconidia size** (X̅): 41.5 x 9.1µm.
Microsporum canis			
GT12	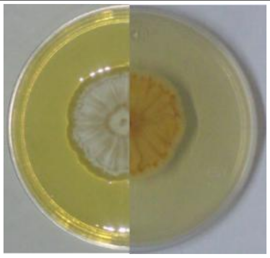	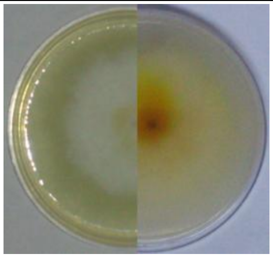	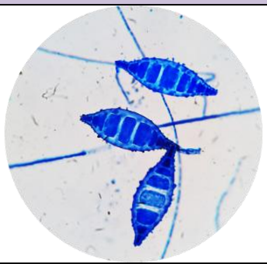
	**Obverse**: colony white, umbilicate, with velvety surface, with radial growth, limited and irregular border.<br><br>**Reverse**: slight production of brown pigment, not diffusible in the medium.	**Obverse**: white colony, cottony surface, with radial growth, limited and irregular border.<br><br>**Reverse**: slight production of yellow-orange pigment, not diffusible in the medium.	Septate mycelium with few equinulate fusiform macroconidia, thick-walled, with 6 to 10 locules; few pyriform microconidia.<br><br>**Microconidia size** (X̅): 4.2 x 1.5µm.<br>**Macroconidia size** (X̅): 73.9 x 22.1µm.
GT14	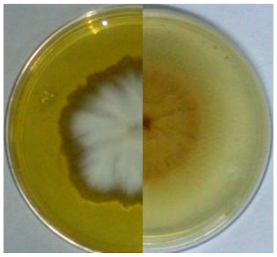	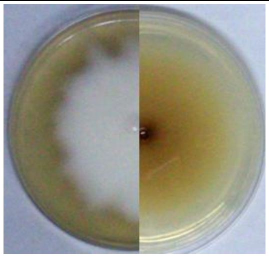	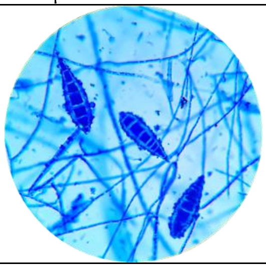
	**Obverse**: white colony, with a floccose surface, with radial growth, limited and irregular border.<br><br>**Reverse**: slight production of brown pigment, not diffusible in the medium.	**Obverse**: white colony, cottony surface, with radial growth, limited and irregular border.<br><br>**Reverse**: slight production of light brown pigment, not diffusible in the medium.	Septate mycelium with few equinulate fusiform macroconidia, thick-walled, with 6 to 8 locules.<br><br>**Macroconidia size** (X̅): 48.1 x 12.6µm.

GT20	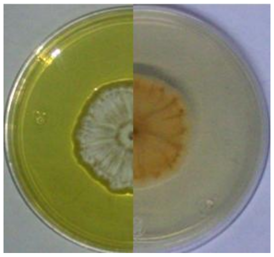	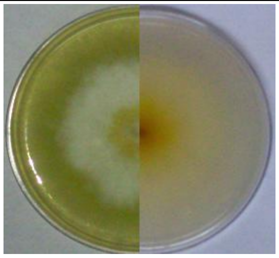	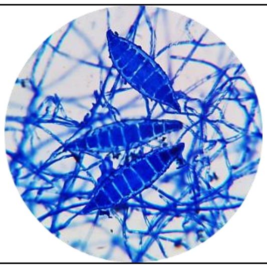
	**Obverse**: white colony, umbilicate, with velvety surface, with radial growth, limited and irregular border.<br><br>**Reverse**: slight production of brown pigment, not diffusible in the medium.	**Obverse**: ocher colony, cottony surface, with radial growth, limited and irregular border.<br><br>**Reverse**: slight production of yellow-orange pigment, not diffusible in the medium.	Septate mycelium with few equinulate fusiform macroconidia, thick-walled with 6 to 10 locules; scarce presence of pyriform microconidia.<br><br>**Microconidia size** ($\bar{X}$): 4.2 x 2.0μm.<br>**Macroconidia size** ($\bar{X}$): 67.5 x 21.1μm.
GT53	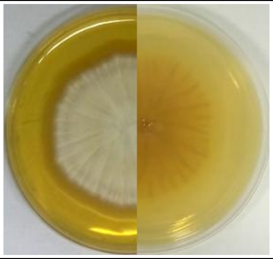	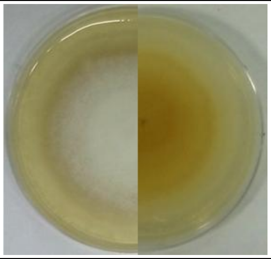	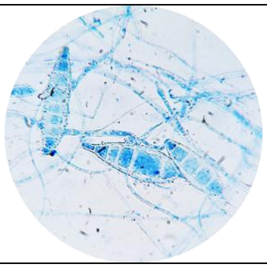
	**Obverse**: white colony, crateriform, with a velvety surface, with radial growth, limited and regular border.<br><br>**Reverse**: pigmentation absent.	**Obverse**: white colony, cottony surface, with radial growth, limited and regular border.<br><br>**Reverse**: slight production of brown-yellow pigment, not diffusible in the medium.	Septate mycelium with abundant spindle-shaped, echinulate, thick-walled macroconidia; with 6 to 10 locules.<br><br>**Macroconidia size** ($\bar{X}$): 57.8 x 13.5μm.
GT56	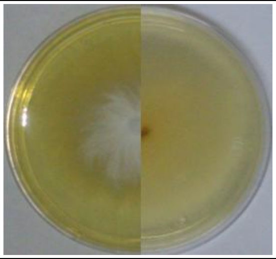	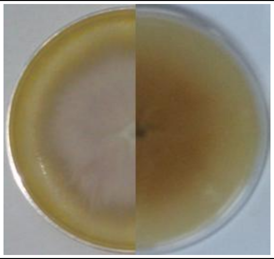	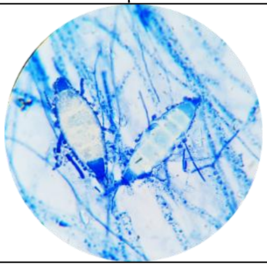
	**Obverse**: white colony, with a floccose surface, with radial growth, limited and irregular border.<br><br>**Reverse**: slight production of	**Obverse**: colony pink, crateriform, floccose surface, with radial growth, limited and irregular border.<br><br>**Reverse**: slight production of light	Septate mycelium with scaate spindle-shaped, equinulate, thick-walled macroconidia with 5 to 9 locules; scarce presence of pyriform microconidia.

	yellow pigment, not diffusible in the medium.	brown pigment, not diffusible in the medium.	**Microconidia size** (X̅): 4.0 x 1.4μm.<br>**Macroconidia size** (X̅): 53.7 x 20.5μm.
GT60	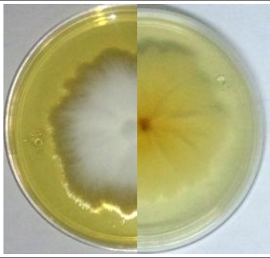	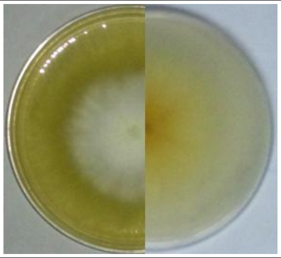	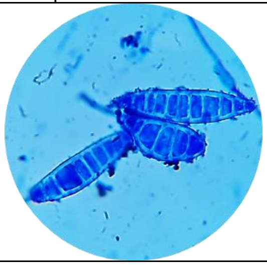
	**Obverse**: white colony, with a floccose surface, with radial growth, limited and irregular border.<br><br>**Reverse**: slight production of yellow pigment, not diffusible in the medium.	**Obverse**: white colony, with a floccose surface, with radial growth, limited and regular border.<br><br>**Reverse**: slight production of yellow pigment, not diffusible in the medium.	Septate mycelium with few fusiform equinulate macroconidia, thick-walled, with 7 to 11 locules.<br><br>**Macroconidia size** (X̅): 53.6 x 15.3μm.
GT61	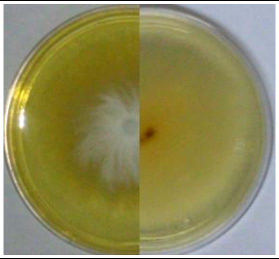	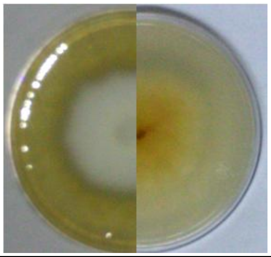	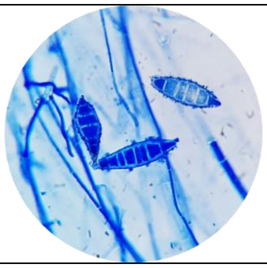
	**Obverse**: white colony, with a floccose surface, with radial growth, limited and irregular border.<br><br>**Reverse**: slight production of yellow pigment, not diffusible in the medium.	**Obverse**: white colony, cottony surface, with radial growth, limited and regular border.<br><br>**Reverse**: slight production of yellow pigment, not diffusible in the medium.	Septate mycelium with few equinulate fusiform macroconidia, thick-walled, with 6 to 8 locules; scarce presence of pyriform microconidia.<br><br>**Microconidia size** (X̅): 3.7 x 1.2μm.<br>**Macroconidia size** (X̅): 48.8 x 16.2μm.
GT64	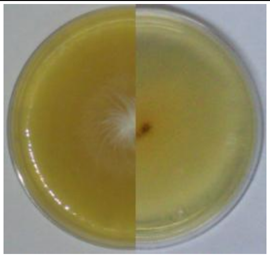	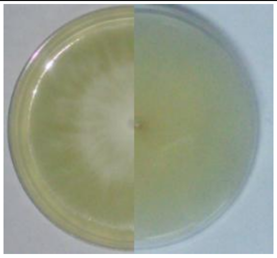	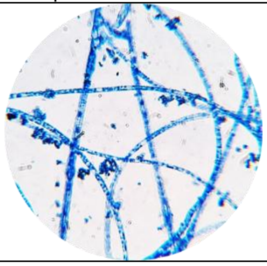
	**Obverse**: ocher-colored colony, with a floccose surface, with	**Obverse**: white colony, with a floccose surface, with radial	Sterile coenocytic mycelium.

	radial growth, limited and irregular border.<br><br>**Reverse**: slight production of yellow pigment, not diffusible in the medium.	growth, limited and regular border.<br><br>**Reverse**: pigmentation absent.	
GT66	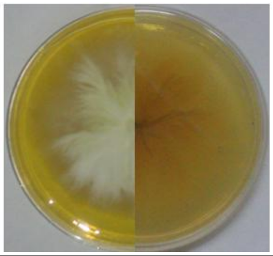	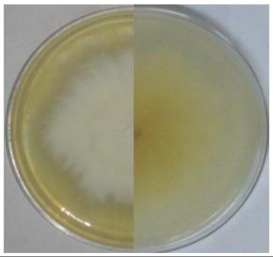	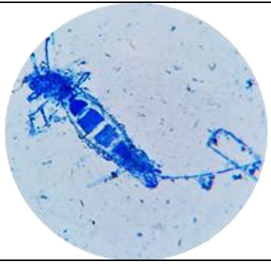
	**Obverse**: white colony, with a floccose surface, with radial growth, limited and irregular border, pale yellow-white color.<br><br>**Reverse**: pigmentation absent.	**Obverse**: pale yellow-white colony, with a floccose surface, with radial growth, limited and irregular border.<br><br>**Reverse**: pigmentation absent.	Septate mycelium with few equinulate fusiform macroconidia, thick-walled, with 4 to 10 locules.<br><br>**Macroconidia size** ($\bar{X}$):<br>49.8 x 18.0µm.
Microsporum nanum			
GT01	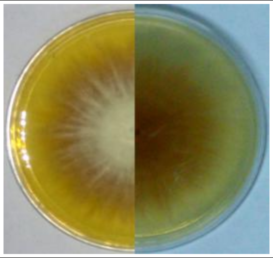	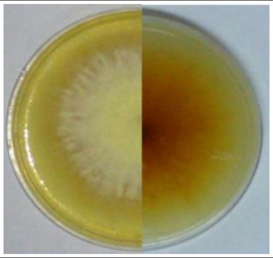	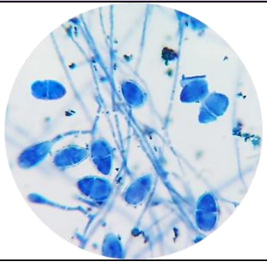
	**Obverse**: pale yellow-white colony, with a powdery surface, with radial growth, limited and irregular border.<br><br>**Reverse**: production of brown-yellow pigment, diffusible in the medium.	**Obverse**: pale yellow colony, powdery surface, radial growth, limited and irregular border.<br><br>**Reverse**: production of yellow-orange pigment, diffusible in the medium.	Septate mycelium with abundant oval, thin-walled macroconidia; with 1 to 2 locules.<br><br>**Macroconidia size** ($\bar{X}$):<br>14.7 x 8.9µm.
GT19	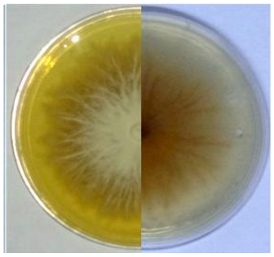	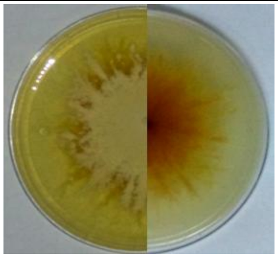	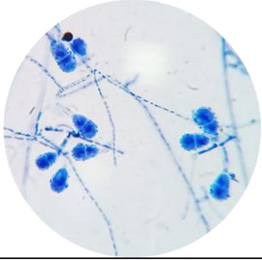
	**Obverse**: pale yellow colony, powdery surface, radial growth, limited and irregular border.	**Obverse**: ocher colony, with a powdery surface, with radial growth, limited and irregular	Septate mycelium with numerous oval, thin-walled macroconidia with 2 locules.

	**Reverse**: production of brown pigment, not diffusible in the medium.	border.<br><br>**Reverse**: production of yellow-orange pigment, diffusible in the medium.	**Macroconidia size** ($\bar{X}$):<br>16.9 x 8.2µm.
GT46	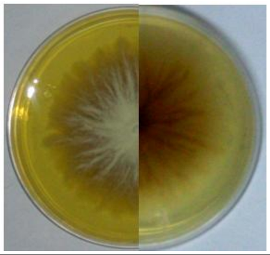	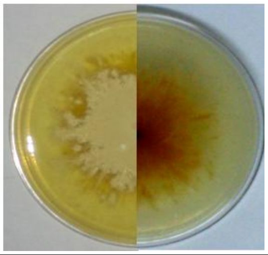	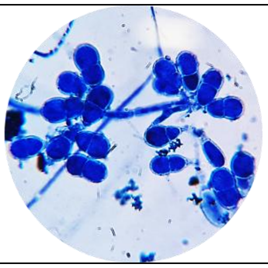
	**Obverse**: pale yellow colony, powdery surface, radial growth, limited and irregular border, yellow.<br><br>**Reverse**: production of brown pigment, not diffusible in the medium.	**Obverse**: ocher colony, with a powdery surface, with radial growth, limited and irregular border.<br><br>**Reverse**: production of yellow-orange pigment, diffusible in the medium.	Septate mycelium with abundant oval, thin-walled macroconidia with 1 to 2 locules.<br><br>**Macroconidia size** ($\bar{X}$):<br>15.8 x 8.4µm.
Epidermophyton floccosum			
MX71	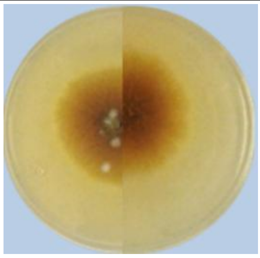	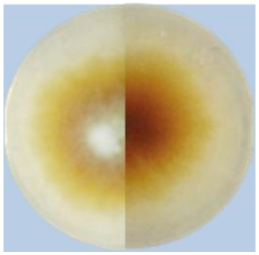	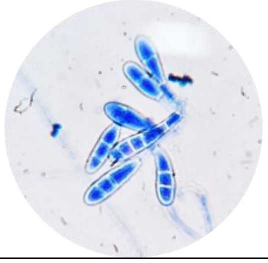
	**Obverse**: yellow colony, umbilicate, with velvety surface, with radial growth, limited and regular border.<br><br>**Reverse**: production of yellow-green pigment, not diffusible in the medium.	**Obverse**: yellow colony, with velvety surface, with radial growth, limited and regular border.<br><br>**Reverse**: production of brown-yellow pigment, not diffusible in the medium.	Septate mycelium with club-shaped macroconidia, with 2 to 4 locules.<br><br>**Macroconidia size** ($\bar{X}$):<br>22.5 x 7.6µm.
