# Supplementary material for: Phenotypic and Genotypic Identification of Dermatophytes from Mexico and Central American Countries
Source: J Fungi (Basel). 2023 Apr 11;9(4):462. doi: 10.3390/jof9040462 (PMC10143779; doi:10.3390/jof9040462)
Supplement: Supplementary file 1 [file Supplementary material-Figure S4.pdf › Supplementary material-Figure S4.pdf]

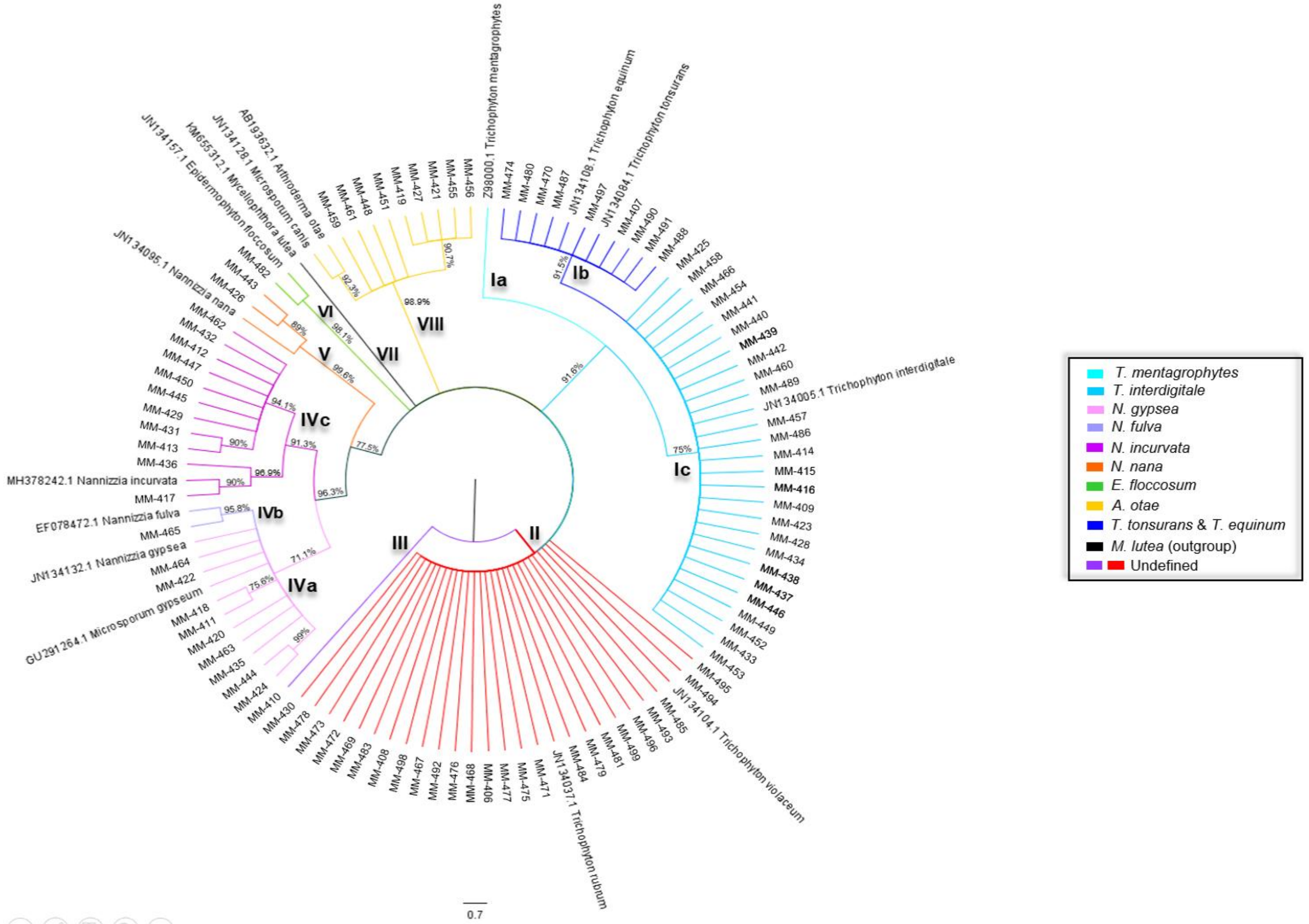

Supplementary Figure S4. Phylogenetic tree of dermatophytes built with sequences from ITS region using the maximum likelihood, trough the MEGAX program. Bootstrap support values are displayed in nodes.
